# 1,3-Benzodioxole Derivatives Improve the Anti-Tumor Efficiency of Arsenicals

**DOI:** 10.3390/ijms23136930

**Published:** 2022-06-22

**Authors:** Xue-Min Shi, Wen-Yan She, Ting-Ting Liu, Lian-Xun Gao, Yu-Jiao Liu, Yi Liu

**Affiliations:** 1 College of Chemistry and Molecular Science, Wuhan University, Wuhan 430072, China; xueminshichem@whu.edu.cn (X.-M.S.); wenyanshe@whu.edu.cn (W.-Y.S.); lxgaochem@whu.edu.cn (L.-X.G.); 2 State Key Laboratory of Separation Membranes and Membrane Processes, School of Chemistry, Tiangong University, Tianjin 300387, China; 2120020069@tiangong.edu.cn

**Keywords:** organic arsenicals, TrxR, ROS, docking, 4T1 tumor, stiripentol

## Abstract

Arsenicals have been widely used in the treatment of cancers such as leukemia and other tumors. However, their side effects limit their clinical application. Stiripentol, a second-line adjunctive treatment for epilepsy with a good safety profile, inhibits microsomal cytochrome-P450-family enzymes to extend the retention time of co-administration. Inspired by the metabolism of stiripentol, the 1,3-benzodioxole responsible for the inhibition and its metabolic derivatives were conjugated with arsenical precursors. The fabricated arsenicals were eliminated much slower in mice and maintained an efficient concentration in the blood for a longer time than that of the arsenical precursors. They also performed better in anti-proliferation by inhibiting the thioredoxin system to induce oxidative stress, and concomitantly to initiate apoptosis in vitro and in vivo. The fabricated arsenicals reversed the hemogram of tumor-bearing mice to normal and eliminated the tumor without causing damage to any organs, exhibiting a good design strategy and pre-clinical application for leukemia and other tumors.

## 1. Introduction

The thioredoxin system, including NADPH, thioredoxin reductase (TrxR), and thioredoxin (Trx), is highly conserved from prokaryotes to humans [1,2]. TrxR belongs to the pyridine nucleotide–disulfide oxidoreductases, and consists of two FAD binding site, two NADPH binding site, and two active catalytic sites. Electrons from the NADPH are transferred to the FAD and then to the N-terminal disulfide. This is followed by transfer to the C-terminal active site of the other monomer of the TrxR dimer complex. The C-terminal active site with 5′-GCUG-3′ is the main reaction site for its substrates, such as Trx. By controlling the exchange of disulfide and thiols, the Trx system regulates the structure and function of its substrates and concomitantly involves several signal pathways, such as transcription, proliferation, anti-oxidation, and apoptosis [3].

According to clinical trials, the Trx system has a strong correlation with lung cancers, diabetes, and pancreatic cancers (ClinicalTrials.gov (accessed on 8 May 2022)). Plenty of studies have revealed that the Trx system is over-expressed in leukemia and tumors compared with the expression in the adjacent normal tissues, such as breast, colorectal, hepatocellular, gastric carcinoma, lung, and pancreatic tissues [4,5,6]. The over-expressed Trx system is widely involved in anti-oxidation, anti-apoptosis, drug tolerance, and even cancer relapse [7].

TrxR is a major regulator of metabolism in leukemia cells by directly regulating GAPDH, altering the glycolysis pathway and redirecting substrates to the pentose phosphate pathway [8]. Implicated by accumulating research, aberrant ROS promote or maintain the oncogenic phenotype by triggering damage to the DNA in myeloid leukemogenesis [9,10]. AML up-regulates its anti-oxidative systems, such as the GSH and Trx systems, to balance the aberrant ROS. In addition, Trx blocks apoptosis [11,12] by binding to the apoptosis-signal-regulating kinase 1 (ASK1) [13], or by regulating caspase 3 activity. Therefore, the Trx system protects cells from ROS before leukemogenesis, while also promoting a shorter relapse interval for the apoptosis resistance induced by drugs during and after leukemogenesis [14]. Therefore, it is necessary to develop inhibitors of the Trx system for cancer therapies.

Inhibitors of the Trx system, especially of TrxR, are widely found among natural and synthesized compounds. Natural inhibitors always have an electrophilic site for which Sec (selenocysteine) has a high affinity, causing irreversible inhibition by covalent binding. The effectiveness of synthetic inhibitors depends on the reaction of selenium with metal elements, such as gold and platinum, and non-metal elements, such as sulfur, selenium, arsenic, etc., according to hard-soft-acid-base theory [15,16,17]. Among these inhibitors, organic arsenicals exhibit high selectivity and efficiency for TrxR. Most of them bind to the C-terminal Sec/Cys pair of TrxR due to the high reactivity of –SeH in the physiological environment [18,19,20]. In spite of their good inhibitory efficiency for TrxR, their side effects limit their clinical application. In order to reach an efficient drug concentration in the blood, clinic doctors usually administer a high single dose or maintain a high administration frequency, which causes severe side effects and thus limits their clinical application [19]. An extension of their retention time in vivo can reduce the arsenical dosage and concomitantly avoid the potential side effects.

Stiripentol is used as the second line of treatment for Dravet syndrome, as an adjunctive treatment when seizures are inadequately controlled by first-line treatment [21,22,23]. Stiripentol inhibits the activity of CYP 1A2 and 3A4 in vivo, thereby extending the retention of co-administration [24]. The interaction of stiripentol and CYPs is mainly attributed to the methylenedioxyphenyl ring system [25]. Furthermore, stiripentol exhibits a good safety and tolerability profile. The inhibition of liver enzyme CYPs by stiripentol is not significantly related to drug-induced injury to the liver or other organs [26,27]. Microsomal cytochrome-P450-family enzymes, which are expressed in the human liver, play vital roles in a wide range of substrate bio-transformations, such as xenobiotic detoxication and procarcinogen bioactivation.

By learning from the mechanisms and metabolism of stiripentol, the methylenedioxyphenyl ring (or 1,3-benzodioxole) and its metabolic derivatives were conjugated with arsenical precursors to produce organic arsenicals. The synthesized organic arsenicals showed a strong inhibition towards the up-regulated Trx system of cancer cells and eliminated tumors by only two administrations once a week without side effects, predicting a good application of arsenicals for the treatment of leukemia and other tumors.

## 2. Results

### 2.1. Arsenicals Were Conjugated with 1,3-Benzodioxole Derivatives

**Scheme 1:**
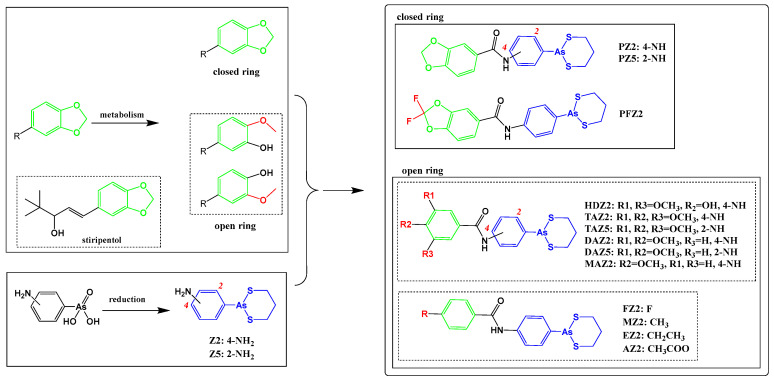
The ring-opening scheme of 1,3-benzodioxole, the conjugation of arsenical precursors, and the related 1,3-benzodioxole derivatives.

**Scheme 2:**
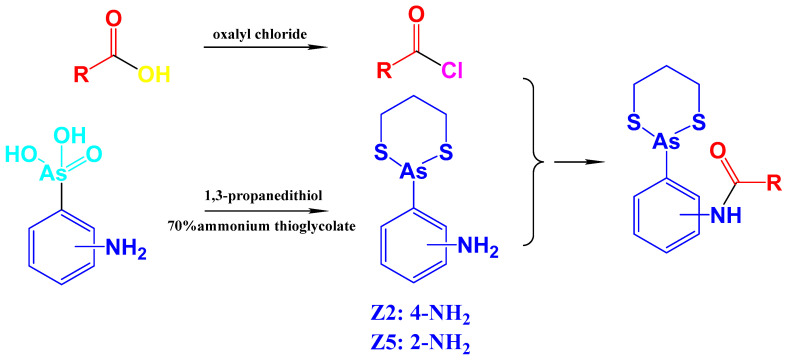
The conjugation of the arsenical precursors and the 1,3-benzodioxole derivatives.

Stiripentol is a second-line anticonvulsant drug, and its metabolites from ring opening also demonstrate anticonvulsant activity [28]. By learning from the metabolism of stiripentol, the related structures were introduced to arsenicals (Scheme 1 and Scheme 2, and Figure S1). 1,3-Benzodioxole either kept its structure or derived to hydroxyl-substituted structures. The arsenicals were synthesized from As(V) precursors, which were mainly produced by ammonium thioglycolate reduction and 1,3-propanedithiol conjugation [19,29].

Firstly, the unchanged structure 1,3-benzodioxole was conjugated with arsenical precursors (Z2 and Z5) through amide bonds, forming PZ2 and PZ5. Considering the cabbeen formation, which further reacts with many substrates to form conjugates during metastasis, fluorine was introduced to the cabbeen-forming site (PFZ2).

Secondly, after ring opening, hydroxyl-substituted structures formed and the structures became more hydrophilic, which supported fast elimination. The ring-opening structures, with single, double, and triple methoxyl(s), were conjugated to explore the metabolism of 1,3-benzodioxole, which produced the HD-, TA-, DA-, and MA-series compounds.

Thirdly, the electron effects of the phenyl were taken into consideration. The electron-withdrawing groups, such as –F and –OOCCH_3_, the electron-donating groups, such as –OCH_3_, and the weak-electron-effect groups, such as methyl and ethyl, were introduced. All the structures of these compounds are shown in Scheme 1 and Scheme 2.

### 2.2. Conjugated Arsenicals Possessed Good Anti-Proliferation Activity

The bioactivity of all the synthetic compounds was evaluated by a MTT assay (Table 1, Tables S1 and S2) against two leukemia cell lines, Molm-13 and NB4; two solid tumor cell lines, HeLa (human cervix cell line) and 4T1 (breast cancer cell line); and two normal cell lines, COS-7 (African green monkey kidney cell line) and rat PBMC (peripheral blood mononuclear cell line).

Firstly, all the organic arsenicals exhibited broad spectrum anti-proliferation efficiency against the above four cancer cell lines, but far less inhibition against the two normal cell lines. The 1,3-benzodioxole derivative showed no anti-proliferation activity to cancer cells, even at a high dosage (Figure S2). Compared with the 1,3-benzodioxole conjugate (PZ2), the fluorine introduction (PFZ2) improved the anti-proliferation activity slightly and metabolized the structures of 1,3-benzodioxole, namely HDZ2, TAZ2, DAZ2, and MAZ2, shared the activity superiority of PFZ2. By comparing MZ2, EZ2 and AZ2 with MAZ2, it was observed that the electron effect had little impact on the total anti-cancer ability, indirectly proving the essential role of the single methoxyl.

The Z2 conjugates were weaker than the Z5 conjugates in anti-cancer proliferation, but the Z2 conjugates possessed a stronger selectivity among cancer cells and normal cells than the Z5 conjugates. For example, the ratio of the *IC*_50_ of TAZ2 on the Molm-13 cells to the *IC*_50_ of TAZ2 on the COS-7 cells was 5.7; that of TAZ5 was 2.8, that of DAZ2 was 7.9, and that of DAZ5 was 3.6. Among these compounds, PFZ2, HDZ2, TAZ2, DAZ2, and MAZ2 had the least influence on normal cell proliferation. Their impact on COS-7 and PBMC did not produce an obvious difference. In addition, MAZ2 had the most effective inhibition on all cancer cell lines, implied by the fact that all its *IC*_50_ values were less than 1 µM. MAZ2 had almost the weakest inhibition on PBMC, further proving its high selectivity between cancer cells and normal cells. When taking all these results together, MAZ2 was selected as the sample compound for further investigation.

### 2.3. MAZ2 Inhibited TrxR Activity by Binding to the C-Terminal Sec/Cys Pair

The vicinal dithiol motif plays a critical role in the structures and functions of proteins by regulating intracellular redox homeostasis, and is responsible for many diseases [30]. Emerging evidence supports the idea that trivalent arsenicals cyclize with vicinal-dithiol-containing proteins, forming stable cyclic compounds [31,32].

**Figure 1:**
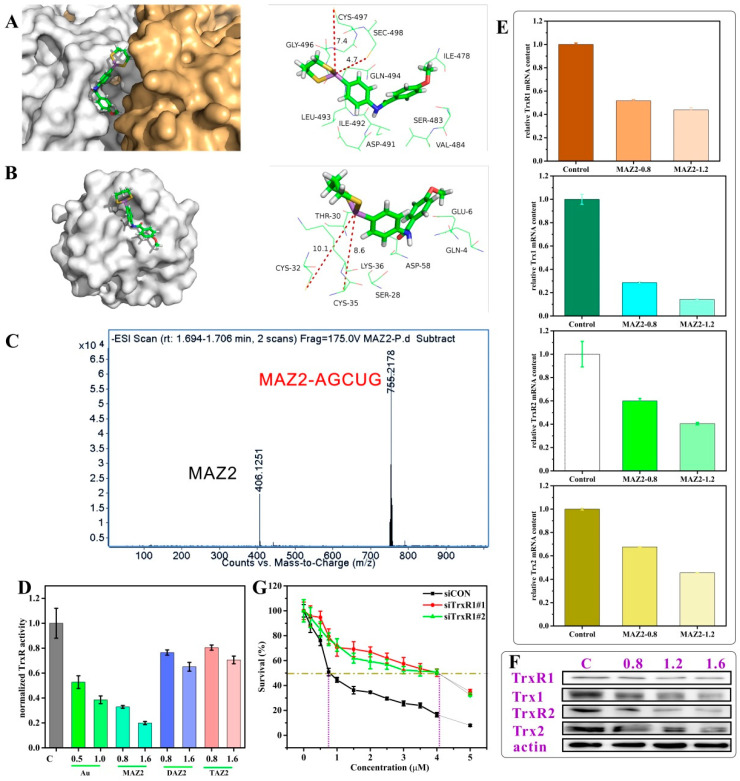
The arsenicals inhibited TrxR activity by binding to the C–terminal Sec/Cys pair; (**A**,**B**) show the binding site of MAZ2 in TrxR1 (PDB: 3EAN) and Trx1 (PDB: 1ERT); (**C**) is an MS spectrum of a mixture of the peptide AGCUG and MAZ2; (**D**) is the TrxR activity of the 4T1 cells after a 12 h incubation of compounds (Au: auranofin); (**E**) is the transcription of the Trx system genes of the 4T1 cells incubated with MAZ2 for 24 h at the dosage of 1*IC*_50_ and 1.5*IC*_50_; (**F**) is the protein content of the Trx system of the 4T1 cells; and (**G**) is an MTT assay of the 4T1 cells with siRNA and MAZ2.

To prove this cyclization, firstly, the binding possibility of arsenicals to TrxR was investigated by docking. As shown in Figure 1A and Figure S3, MAZ2 reached the catalysis site of the C-terminal with a distance to the reactive amino acid residue Sec 498 of 4.7 Å, and a distance to Cys 497 of 7.4 Å. The binding free energy was −5.20 kcal/mol (Table S3), demonstrating a reaction possibility. A hydrogen bond formed between the arsenical and the Asp49 of TrxR (Figure S3C). In the most stable docking configuration of MAZ2 in Trx (Figure 1B and Figure S3), MAZ2 could not insert into the active site of Trx because of the far distance between the arsenic and the active sites. The difference between TrxR and Trx demonstrated the selectivity of MAZ2 between TrxR and Trx. Z2 was much too far from the Sec 498 and Cys 497, and it had a lower binding energy with TrxR (Table S3 and Figure S4). Compared with MAZ2, Z2 also had less selectivity and inhibition for TrxR, implying our good design strategy.

In order to reduce a wide range of substrates, TrxR presents its catalytic site on the protein surface. The C-terminal active sequence, GCUG, possesses the highly reactive groups –SH and –SeH, which can react with arsenic in physiological conditions because the pKa of –SeH is 5.2 [3,33]. According to hard-soft-acid-base theory, –SH has a high affinity for arsenic. Taking all of these together, arsenic could react with the spatially vicinal thiol/selenol pair, forming a covalently binding complex [34,35]. After approaching the active site of TrxR (Figure 1A), MAZ2 reacted with TrxR by conjugating with the Sec 498 and Cys 497, forming a stable cyclic structure. This was confirmed by the conjugates of the synthetic peptide AGCUG, the C-terminal of human TrxR, which was detected by MS spectra (AGCUG–MAZ2, *m/z* = 755.2178; MAZ2, *m/z* = 406.1251) as shown in Figure 1C and Figure S5. The real human C-terminal sequence is SGCUG, but considering the stability of SGCUG, the S was replaced by A; the A had no influence on the reactivity of the active amino acid residues C and U. The molar ratio of AGCUG and MAZ2 was 1:1.2, so there was some MAZ2 left.

TrxR reduces its substrates by thiol–disulphide exchange. If Sec 498 and Cys 497 were covalently bound by arsenicals, TrxR would lose its function permanently. Indeed, the covalent binding of TrxR by MAZ2 led to inactivity of substrate reduction, illustrated by the sharply reduced activity of the 4T1 cellular TrxR (Figure 1D). Auranofin is a clinically used TrxR inhibitor and was used as a positive control [36,37]. MAZ2 performed better than auranofin at its own *IC*_50_, and far better than the DAZ2 and TAZ2 structures, containing two and three methoxyls, respectively, which provided evidence of MAZ2′s stronger anti-cancer proliferation efficiency (Table 1). Interestingly, MAZ2 not only inhibited the activity of TrxR, but also decreased the transcription of the Trx system (Figure 1E). Both the cytosol forms (TrxR1 and Trx1) and the mitochondrial forms (TrxR2 and Trx2) were influenced intensively. The protein contents shared the same trend as the mRNA content, as determined by western blot (Figure 1F).

To further verify the target of MAZ2, TrxR was knocked down by siRNA, as shown by the reduced mRNA and protein contents (Figure S6). Both the siRNAs reduced the TrxR transcription by a large extent. After the TrxR was knocked down, the anti-cancer proliferation efficiency of MAZ2 was dramatically weakened, with *IC*_50_ value changing by 5.5-fold, as shown in Figure 1G.

Emerging research has reported that the Trx system is over-expressed in leukemia and tumors compared with the adjacent normal tissues [4,5,6]. The over-expressed Trx system is widely involved in anti-oxidation, anti-apoptosis, drug tolerance, and even cancer relapse [7]. These differences between tumors and normal tissue provide an interpretation of the selectivity of MAZ2 for cancer cells relative to normal cells (Table 1).

These data collectively suggest that TrxR was the target of MAZ2 and that MAZ2 possessed strong anti-cancer proliferation efficiency by selectively and irreversibly inhibiting TrxR.

### 2.4. ROS Bursting after TrxR Inhibition by Arsenicals

Oxidoreduction reactions and processes pervade biology at several levels: biosensors such as the NAD(P)+/NAD(P)H ratio for metabolic status, oxidoreductases involved in many biochemical reactions, and essential bioenergetic processes based on electron transfer reactions [38]. A redox balance is always broken by bursting ROS, which are the results of reduced free-radical-scavenging enzymes and elevated intracellular oxidant production [39].

**Figure 2:**
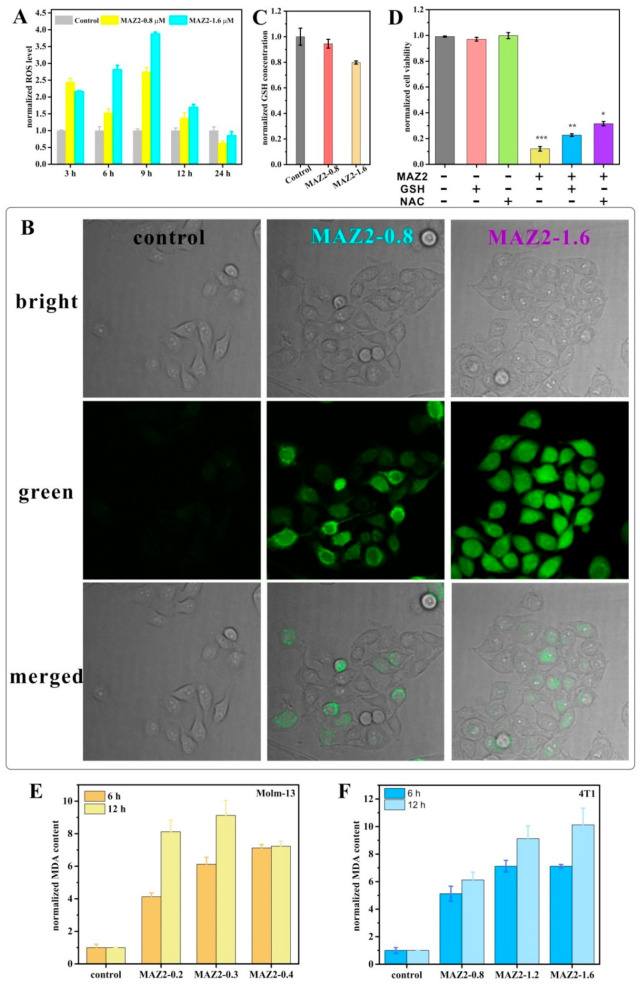
ROS bursting after TrxR inhibition by arsenicals; (**A**) is the fluorescence intensity of the 4T1 cells stained by DCFH-DA (the fluorescence was detected by flow cytometry); (**B**) is the 4T1 cells stained by DCFH-DA (the fluorescence was detected by confocal); (**C**) is the GSH contents; (**D**) is the cell viability under MAZ2 treatment without GSH or NAC; and (**E**,**F**) are the MDA contents of the Molm-13 and 4T1 cells. Significance was set at * *p* < 0.05, ** *p* < 0.01 and *** *p* < 0.001.

Small antioxidant metabolites, antioxidant enzymes, and damage repair enzymes work as the three most important defences against oxidative stress. The most essential antioxidant enzymes are the GSH system and the Trx system. The Trx system scavenges ROS either by direct reactions or by synergistically working with other partners. After TrxR was inhibited by MAZ2 in this study, the ROS level rose sharply in a dosage-dependent manner (Figure 2A), which was monitored by a green fluorescent dichlorodihydrofluorescein diacetate (DCFH-DA) metabolite (a widely used ROS fluorescent sensor). Obvious ROS could be detected after 3 h of incubation with MAZ2, and the peak was reached after 9 h.

To further confirm the dramatically elevated ROS levels, DCFH-DA-related signals were used to monitor the ROS levels of the 4T1 cells. As shown in Figure 2C, faint fluorescence was present in cells of the control group (Figure 2B). After 9 h of treatment with MAZ2, bright green fluorescence filled the cells, implying a high level of ROS. In addition, the ROS levels increased in a dosage-dependent manner, coinciding with the flow cytometry tests (Figure 2A,B). Toxic levels of ROS are a disaster for cancer cells, inducing cell death. Long-term incubation causes gradual death, which was implied by the slightly round shape in MAZ2-treated groups (Figure 2B), thus indicating fewer cellular ROS contents.

The GSH system is another warrior in the anti-oxidant war. However, MAZ2 had very little influence on the GSH content (Figure 2C). Furthermore, the pre-treatment of GSH does not save cells from MAZ2 damage (Figure 2D). NAC prevents more deaths from MAZ2 than GSH, implying that the ROS play a partial role in the damage of MAZ2, but are not entirely responsible for the damage of MAZ2.

Bursting ROS disrupt biomolecules, such as proteins, nucleic acids, and lipids. MAZ2 incubation led to the peroxidation of lipids in both the Molm-13 and 4T1 cells, as determined by MDA contents. In agreement with the ROS level trends, the MDA content increased acutely and dosage-dependently (Figure 2E,F). All these data support ROS bursting after TrxR inhibition by arsenicals.

### 2.5. Apoptosis was Induced by MAZ2 Incubation

**Figure 3:**
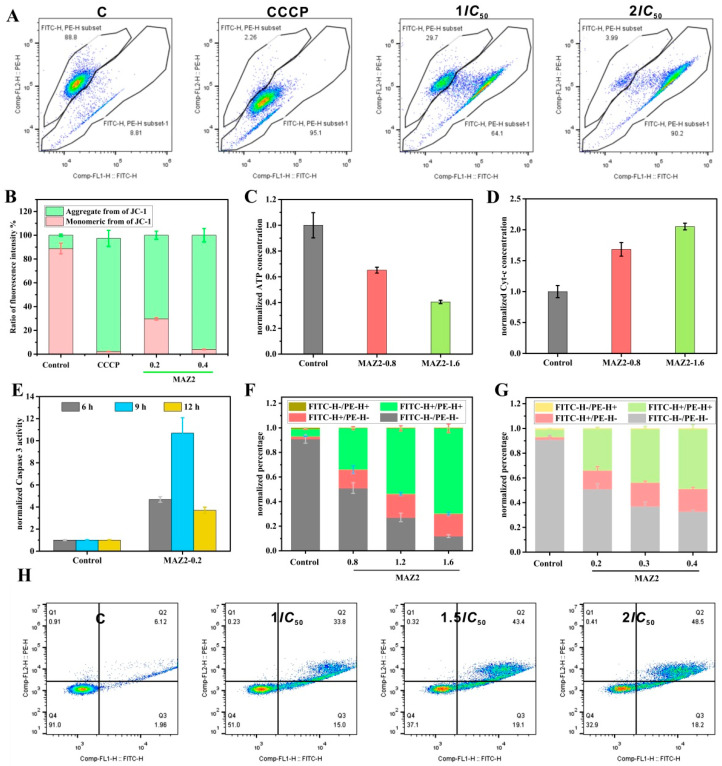
Apoptosis was induced by MAZ2 incubation; (**A**) is the membrane potential monitored by JC-1 staining; (**B**) is the ratio of aggregated JC-1 molecules to the monomeric molecules of (**A**); (**C**) is the ATP level; (**D**) is the cytosol’s cytochrome c content; (**E**) is the caspase 3 activity; (**F**) is the 4T1 cells’ apoptosis assay by flow cytometry; and (**G**,**H**) show the Molm-13 cells’ apoptosis assay by flow cytometry.

Mitochondria and NADPH oxidases are two major contributors of endogenous ROS in cancer. The leakage of electrons from the electron transport chain generates many ROS, and the resulting Trx system strike and redox imbalance may cause mitochondrial breakdown due to ROS-disrupting biomolecules. The inner mitochondrial membrane did not remain intact and gradually collapsed, leading to a reduced membrane potential (Figure 3A). The cells of the control group possessed intact membranes and a high membrane potential. The uncoupling agent CCCP dissipated the proton gradient of the inner membrane and interspace, leading to a completely collapsed membrane potential (Figure 3B). MAZ2 also manifested a strong efficiency for dissipating the proton gradient and thus reducing the ATP content (Figure 3C).

Mitochondrial dysfunction can initiate mitochondria-dependent apoptosis by releasing cytochrome c into the cytosol (Figure 3D), which is determined by caspase-3-cleaved activation. In addition, the trans-nitrosation of caspase 3 by Trx is related to apoptosis evasion [12].

The Trx inhibition by arsenicals can increase the nitrosation of caspase 3 by Trx, which is implied by the dramatically increased activity (Figure 3E). The activated caspase 3 released caspase-activated DNase (CAD) from the inhibitor of CAD, which cleaved the DNA to 180 bp–200 bp. Finally, the cancer cells suffered from apoptosis (Figure 3F–H). MAZ2 induced apoptosis in a dosage-dependent manner.

### 2.6. 4T1 Tumors Were Eliminated by Two Administrations of MAZ2

**Figure 4:**
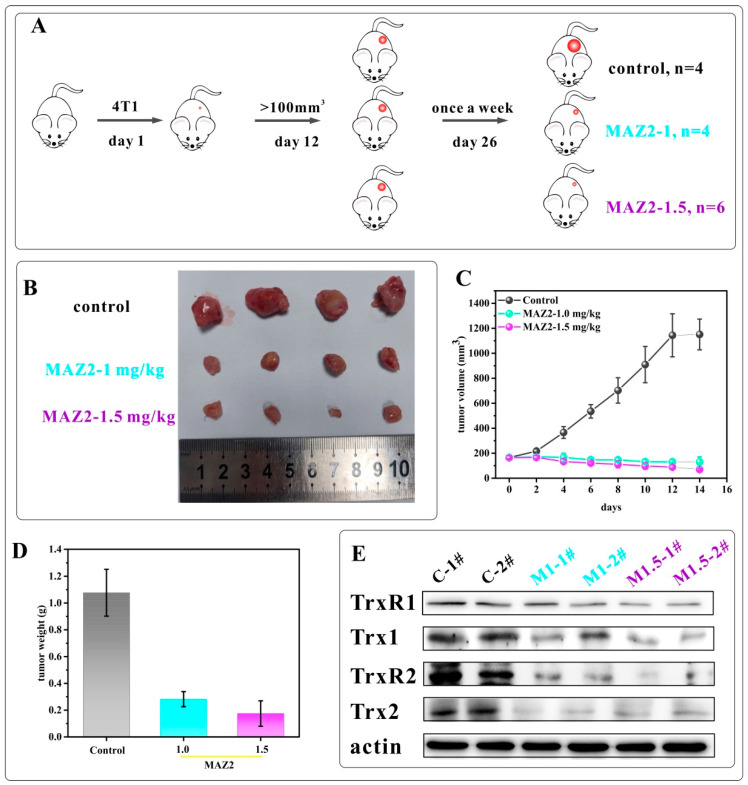
The 4T1 tumors were eliminated by two administrations of MAZ2; (**A**) is a scheme of the mice experiments; (**B**) is a tumor picture after mice sacrifice; (**C**) is the tumor volume; (**D**) is the tumor weight; and (**E**) is the Trx system protein content determined by WB.

To confirm the bioactivity of MAZ2 in vivo, the tumor growth inhibition in 4T1 tumor syngeneic models in Balb/C mice was investigated (Figure 4A). When tumors grew to about 100 mm^3^, the mice were divided into three groups; namely, the control, 1 mg/kg of MAZ2, and 1.5 mg/kg of MAZ2. Either a vehicle or the MAZ2 was injected intraperitoneally once a week. After two weeks, two tumors in the mice belonging to the 1.5 mg/kg MAZ2 group were eliminated, and one was eliminated a few days later (the 3rd one from left to right in Figure 4B). Therefore, the experiments were terminated at day 26 after two administrations.

The tumors in the vehicle (control) group grew rapidly, while an injection of 1 mg/kg or 1.5 mg/kg of MAZ2 once a week could completely reverse the growth of tumors and gradually eliminate tumors (Figure 4C). The average tumor weight of the MAZ2 administration groups was reduced by 30% and 15%, respectively (Figure 4D), supporting the high anti-tumor activity of MAZ2.

To prove the target of MAZ2 in vivo, the TrxR and Trx content was determined by western blot (Figure 4E). As shown in Figure 4E, two tumors in each of the groups were dissolved and the TrxR and Trx content from each tumor of every group demonstrated almost no difference, implying credible results. MAZ2 reduced the TrxR1 content slightly and reduced the Trx1 content by a large amount. The mitochondrial forms of the Trx system proteins were almost eliminated by MAZ2 administration.

**Figure 5:**
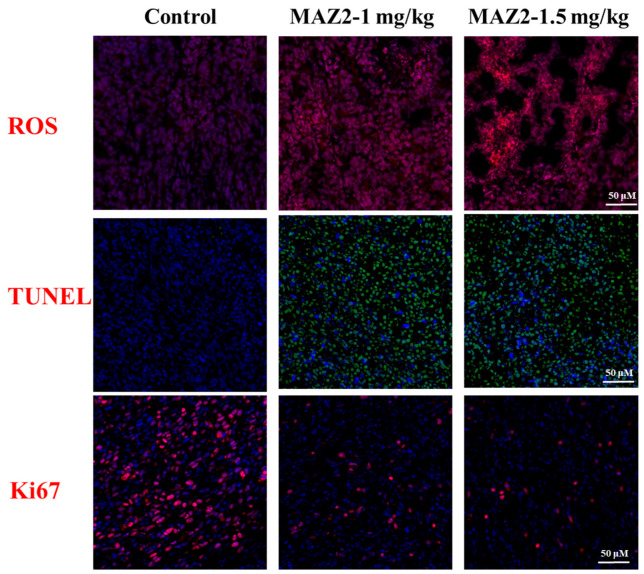
Staining of tumor tissues. ROS: blue-nuclear, red-ROS; TUNEL: blue-nuclear, green-TUNEL; Ki67: blue-nuclear, red-Ki67.

After the inhibition of the Trx system by MAZ2, the ROS level was elevated dramatically (Figure 5). The bursting ROS disrupted tumor cells, leading to apoptosis, which was determined by obvious TUNEL signals. Concomitant with the widely occurring apoptosis, the malignant proliferation potential was largely reduced, as monitored by Ki67 staining.

### 2.7. MAZ2 Administration Had No Obvious Side Effects

**Figure 6:**
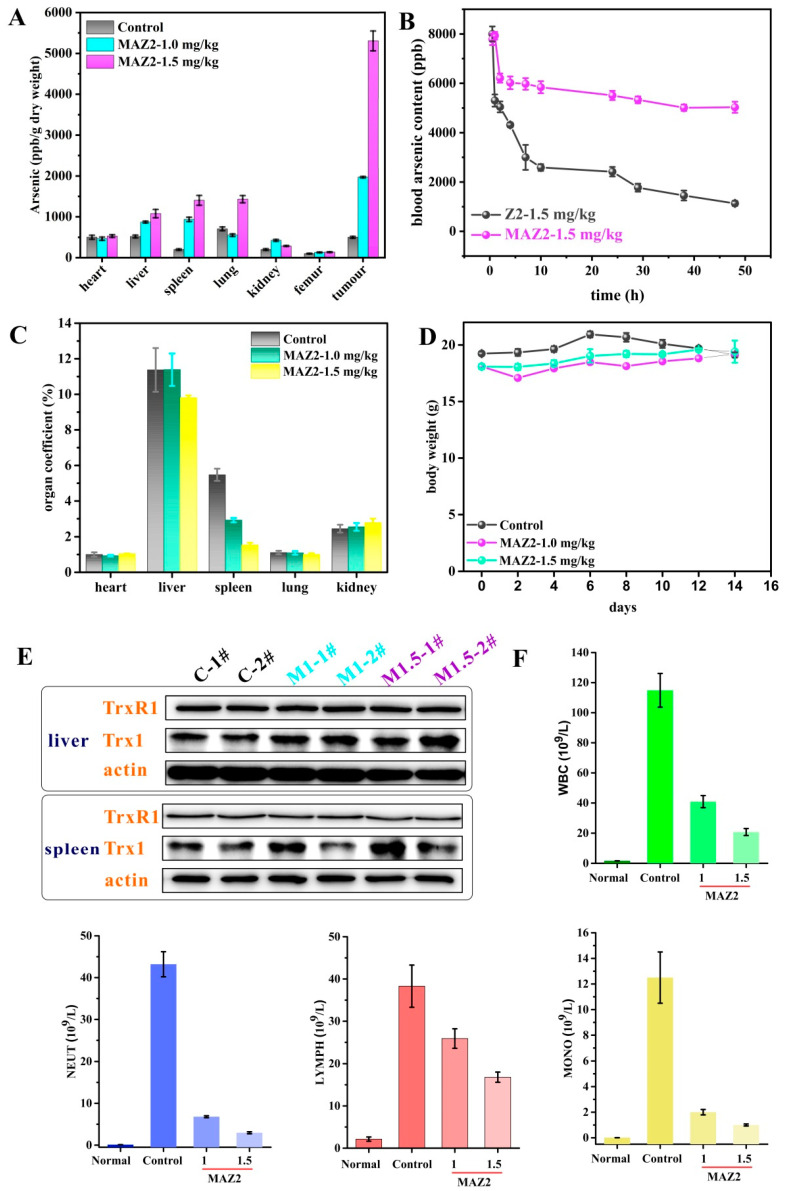
MAZ2 administration had no obvious side effects; (**A**) is the arsenic content; (**B**) is the blood arsenic content; (**C**) is the organ coefficient; (**D**) is the body weight; (**E**) is the TrxR and Trx content of the liver and spleen; and (**F**) is the blood cell counts.

Except for the strong TrxR inhibition, the MAZ2 gathered in the tumor instead of the viscera (implied by high arsenic content in the tumor, as shown in Figure 6A). In general, the viscera arsenic content increased with an increased dosage of MAZ2. The gathering of MAZ2 in the tumor amplified the tumor-eliminating efficiency.

To investigate the reason that an administration only once per week could completely eliminate a tumor, the pharmaceutical kinetics of MAZ2 and its arsenical precursor, Z2, were monitored. As shown in Figure 6B, the blood arsenic content reached their peaks at the same time due to the same dosage of Z2 and MAZ2. The Z2 was eliminated rapidly, and after 48 h, the arsenic content was reduced to a very low level. The arsenic content of the MAZ2 groups decreased slowly and remained at high levels even after 2 days. After 4 days, the MAZ2 groups were still experiencing high arsenic levels (Figure S7), indicating a relatively higher arsenic level and a higher anti-tumor efficiency.

The tumor-bearing mice were always in bad health, as illustrated by an enlarged liver and spleen (Figure 6C). The MAZ2 treatment reduced the damage of the tumor to the viscera, especially the liver and spleen. Both the tumor and the MAZ2 administration had no obvious impact on the body weight of the mice, as shown in Figure 6D.

The liver is the most important organ in metabolism; it is responsible for the biotransformation of a large range of exotic substrates. Considering the strong inhibition of tumor TrxR, the TrxR content of the liver and spleen were tested. From Figure 6E, no changes were exhibited in all samples, demonstrating the selectivity of MAZ2. The blood content of mice from the three groups and normal mice were measured (Figure 6F). Compared with the normal group, mice from the control group had far more blood cells, such as WBC, NEUT, LYMPH, and MONO. The MAZ2 administration lowered the counts of blood cells and reversed abnormalities in the hematological system.

**Figure 7:**
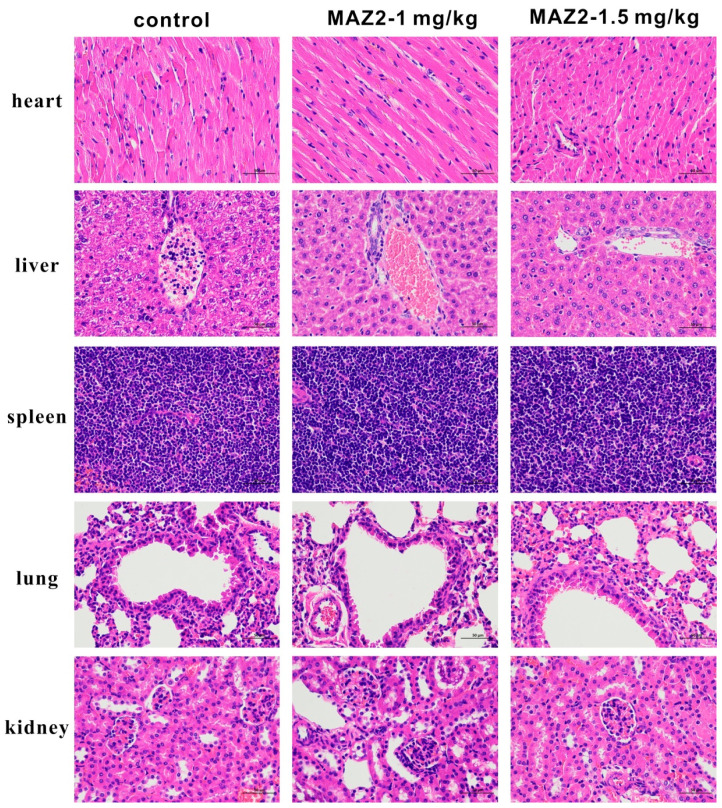
H&E staining of the heart, liver, spleen, lungs, and kidneys from the three groups.

Compared with normal mice, with dense and healthy organs, the tumor-bearing mice without drug intervention had loose and destroyed organs (Figure 7), manifested by nuclear blebbing, cell swelling, and even necrosis—especially in the liver. MAZ2 can block the malignant growth of tumors, thus reducing the damage of the tumor to the organs. Therefore, the H&E staining of the two MAZ2-administered groups showed healthy organs.

## 3. Discussion

ROS are involved in many biological processes, such as enhanced cell proliferation, apoptosis evasion, and tumorigenesis, by interacting with the MAPK (mitogen-activated protein kinase), the PI3K (phosphoinositide 3-kinase), and other signal pathways.

By up-regulating the antioxidant system, cancer cells keep the redox system in balance. In the antioxidant system, the Trx system works through the thiol redox reaction to scavenge ROS and modulating substrate structures and functions via the thiol/disulfide exchange. To control the elevated ROS, the Trx system is over-expressed in a wide range of tumors and blood cancers.

Toxic levels of ROS exhaust the antioxidant system capacity, causing programmed cell death and providing a classic anti-cancer method. Chemotherapy, such as bleomycin, cisplatin, and arsenic trioxide, is widely used in clinics through ROS, which causes irreparable damage and cell death [39]. Given the essential role of the anti-oxidant system, inhibitors of the anti-oxidant system have been widely developed. Arsenic trioxide is a classic ROS-maker and apoptosis-inducer, which has been used as the target drug for acute promyelocytic leukemia. Inspired by the success of arsenic trioxide, organic arsenicals with easily modifiable structures and high bioactivity are of great interest.

According to the hard-soft-acid-base theory, arsenic has a high affinity for thiol and selenol. Thiol is present in most proteins, while there are only about 20 selenium-containing proteins. Considering the activity difference of thiol and selenol, arsenicals bind to selenol first. When taking the approach into account, arsenicals prefer binding with TrxR because the selenol is exposed on the surface, whereas the selenol of other proteins is mainly hidden inside the proteins [3]. Taking these collectively, TrxR is the exclusive target of arsenicals. However, when TrxR is in a relatively low amount, arsenicals can bind with other proteins. This sequential binding order may explain why the knocking down of TrxR only lifts the *IC*_50_ value several folds instead of tens or hundreds of folds. Thus, the dosage should be strictly controlled to manage the side effects of arsenicals.

**Figure 8:**
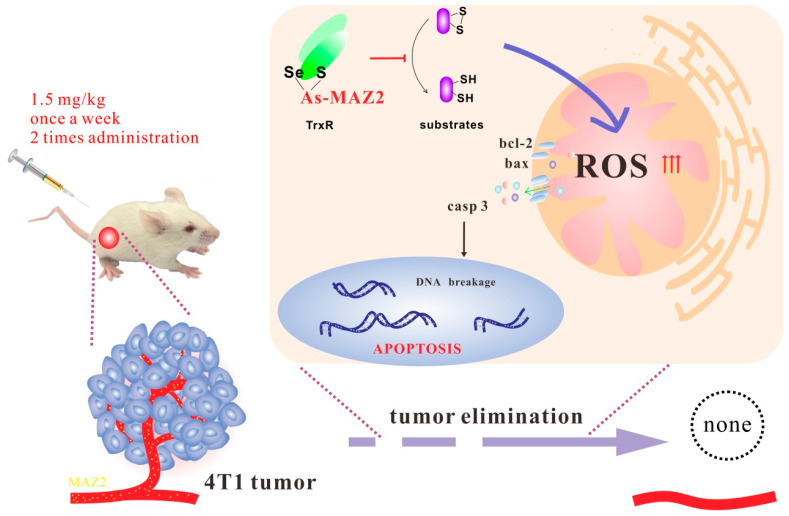
The mechanism of MAZ2.

Here, we developed an organic arsenical, MAZ2, with a high inhibitory effect on TrxR and a high anti-tumor efficiency. MAZ2 inhibited the TrxR by binding to the catalytic site of the C-terminal, causing ROS bursting. The dramatically elevated ROS disrupted biomolecules, especially the energy-producing metabolism center, mitochondria. Apoptosis was initiated by the dysfunction and imbalance of homeostasis (Figure 8).

The dosage of MAZ2 and the administration frequency were relatively lower than that of arsenic trioxide and that of some of our previously developed arsenicals. These arsenicals shared the active structure of arsenic, but had other functional groups. These different functional groups may lead to a different performance in vitro and in vivo. The typical dosage of arsenic trioxide is about 5 mg/kg, which is far larger than our 1 or 1.5 mg/kg (considering the molecular concentration) [40]. In addition, the administration frequency was twice a week or every two days, in general [29]. Here, MAZ2 was administrated once a week and only administered twice overall to eliminate tumors. First of all, these results indeed prove the high anti-tumor efficiency of MAZ2. From another aspect, is there any possibility that a high dosage of arsenic inhibits the anti-tumor functions of the mice themselves, such as the immune system? This hypothesis needs further investigation.

## 4. Materials and Methods

### 4.1. Chemicals and Instruments

Magnetic resonance (^1^H and ^13^C NMR) spectra were recorded on a Bruker Avance III HD 400 Hz spectrometer; LC-MS assays were conducted using an Agilent 1260 Infinity II liquid chromatograph and an Agilent 6530 Q-TOF mass spectrometer.

### 4.2. Synthesis and Characterization of Z2 and Z5

The synthesis was performed following the protocol described in our previous work.

**Z2:** 4-(1,3,2-dithiarsinan-2-yl)aniline. Yield: 2.62 g (52.1%). ^1^H NMR (400 MHz, CDCl_3_) δ: 7.66–7.64 (d, *J* = 8.0 Hz, 2H), 6.79–6.77 (d, *J* = 8.0 Hz, 2H), 3.86 (s, NH_2_, 2H), 2.93–2.87 (m, 2H), 2.75–2.69 (m, 2H), 2.19–2.10 (m, 1H), 1.97–1.89 (m, 1H). ^13^C NMR (400 MHz, CDCl_3_): 147.54, 133.81, 125.73, 115.65, 28.72, 26.44. ESI-MS: calculated for C9H12NS2As: 272.9627, found 273.9708[M + H]^+^.
**Z5:** 2-(1,3,2-dithiarsinan-2-yl)aniline. Yield: 1.94g (40.1%). ^1^H NMR (400 MHz, DMSO-d6) δ: 7.62–6.60 (d, *J* = 8 Hz, 2H), 7.19–7.15 (m, 1H), 6.76–6.70 (m, 2H), 5.45 (s, NH_2_, 2H), 2.94–2.91 (m, 4H), 2.08–2.00 (m, 1H), 1.92–1.83 (m, 1H). ^13^C NMR (400 MHz, DMSO-d6): 151.63, 134.66, 131.79, 118.42, 117.83, 116.59, 28.95, 27.84. ESI-MS: *m/z* calculated for C9H12NS2As: 272.9627, found 273.9700[M + H]^+^.

### 4.3. Synthesis and Characterization of Conjugates of Arsenical Precursors and 1,3-Benzodioxole Derivatives

PZ2 was set as the example. Piperonylic acid (750 mg, 5 mM) was dissolved in dry CH_2_Cl_2_ (20 mL) under the protection of nitrogen; then, 635 μL oxaloyl chloride and N, N-dimethylformamide (DMF) in a catalytic amount were added, followed by 40 min of stirring. Next, the mixture was dried to obtain a light yellow solid, benzo[d][1,3]dioxole-5-carbonyl chloride, without post-treatment. It was directly used for the next step.

In an ice bath, 830 mg (4.5 mM) of piperyl chloride was dissolved in 10 mL of dichloromethane under nitrogen protection; then, 820 mg of Z2 (dissolved in 15 mL of dichloromethane) was added to the solution, followed by adding 500 μL of pyridine or triethylamine. The reaction progress was monitored by thin layer chromatography. The reaction was stopped when the band of piperyl chloride disappeared in thin layer chromatography. The reaction usually finished in 2–3 h. The reaction solution was washed by 10% HCl once, and the organic phase was separated and purified by silica gel column chromatography (silica: DCM/PE = 2/1, V/V).

**PZ2:** N-(4-(1,3,2-dithiarsinan-2-yl)phenyl)benzo[d][1,3]dioxole-5-carboxamide (P: Piperonylic acid). Yield: 644 mg (51%). ^1^H NMR (400 MHz, DMSO) δ: 10.25 (s, NH, 1H), 7.95–7.93 (m, 2H), 7.80–7.77 (m, 2H), 7.60–7.58 (d, *J* = 8.0 Hz, 1H), 7.53 (s, 1H), 7.08–7.06 (d, *J* = 8.0 Hz, 1H), 6.14 (s, 2H), 2.84–2.73 (m, 4H), 2.03–1.89 (m, 2H). ^13^C NMR (400 MHz, DMSO): 165.19, 150.67, 147.88, 140.86, 133.13, 132.21, 128.96, 123.37, 121.32, 108.45, 108.24, 102.34, 28.36, 26.15. ESI-MS: *m/z* calculated for C_17_H_16_AsNO_3_S_2_ [M]: 420.9788; [M – H]^−^: 419.9715; found 419.9713.
**PZ5:** N-(2-(1,3,2-dithiarsinan-2-yl)phenyl)benzo[d][1,3]dioxole-5-carboxamide (P: Piperonylic acid). Yield: 505 mg (40%). ^1^H NMR (400 MHz, DMSO) δ: 10.46 (s, NH, 1H), 8.10–8.08 (d, *J* = 8 Hz, 1H), 7.52–7.49 (m, 2H), 7.42–7.35 (m, 2H), 7.08–7.06 (d, *J* = 8 Hz, 1H), 2.94–2.87 (m, 2H), 2.82–2.76 (m, 2H), 2.05–1.75(m, 2H). ^13^C NMR (400 MHz, DMSO): 165.67, 150.75, 147.88, 140.64, 143.79,143.17, 130.92, 128.16, 126.79, 126.22, 123.62, 108.51, 108.21, 102.34, 29.17, 28.18. ESI-MS: *m/z* calculated for C_17_H_16_AsNO_3_S_2_ [M]: 420.9793; [M + H]^+^: 421.9860; found 421.9856; [2M + Na]^+^: 864.9467; found 864.9459.
**MAZ2:** 2-(4-(1,3,2-dithiarsinan-2-yl)phenyl)-1-(4-methoxyphenyl)ethan-1-one (MA: 4-methoxybenzoic acid). Yield: 105 mg (35%). ^1^H NMR (400 MHz, DMSO) δ: 10.29 (s, NH, 1H), 8.00–7.95 (m, 4H), 7.80–7.78 (d, *J* = 8 Hz, 2H), 7.09–7.07 (d, *J* = 8 Hz, 2H), 3.85 (s, 3H), 2.85–2.74 (m, 4H), 2.05–1.89 (m, 2H). ^13^C NMR (400 MHz, DMSO): 135.45, 134.14, 134.04, 130.81, 130.69, 119.40, 118.55, 115.40, 55.0, 18.96, 14.88. ESI-MS: *m/z* calculated for C_17_H_18_AsNO_2_S_2_ [M]: 407.0021; [M – H]^−^: 405.9922; found 405.9975.
**DAZ2:** N-(4-(1,3,2-dithiarsinan-2-yl)phenyl)-3,4-dimethoxybenzamide (DA: 3,4-dimethoxybenzoic acid). Yield: 206 mg (40%). ^1^H NMR (400 MHz, DMSO) δ: 10.27 (s, NH, 1H), 7.97–7.94 (d, *J* = 8 Hz, 2H), 7.81–7.79 (d, *J* = 8 Hz, 2H), 7.67–7.64 (s, 1H), 7.56–7.55 (s, 1H), 7.12–7.10 (d, *J* = 8 Hz, 2H), 3.86 (s, 6H), 2.85–2.74 (m, 4H), 2.03–1.90 (m, 2H). ^13^C NMR (400 MHz, DMSO): 165.60, 152.26, 148.80, 140.93, 133.12, 132.14, 127.22, 121.42, 111.56, 111.38, 56.17, 56.11, 28.38, 26.18. ESI-MS: *m/z* calculated for C_18_H_20_AsNO_3_S_2_ [M]: 437.0018; [M – H]^−^: 436.0028; found 436.0024.
**DAZ5:** N-(2-(1,3,2-dithiarsinan-2-yl)phenyl)-3,4-dimethoxybenzamide (DA: 3,4-dimethoxybenzoic acid). Yield: 123 mg (42%). ^1^H NMR (400 MHz, DMSO) δ: 10.47 (s, NH, 1H), 8.11–8.09 (d, *J* = 8 Hz, 1H), 7.64–7.62 (d, *J* = 8 Hz, 1H), 7.54–7.49 (m, 2H), 7.42–7.37 (m, 2H), 7.12–7.10 (d, *J* = 8 Hz, 1H), 3.85 (s, 6H), 2.95–2.89 (m, 2H), 2.84–2.78(m, 2H), 2.04–2.00(m, 1H), 1.84–1.75 (m, 2H). ^13^C NMR (400 MHz, DMSO): 166.07, 152.08, 148.62, 140.77, 134.30, 130.98, 126.20, 121.81, 111.50, 56.18, 56.11, 29.22, 28.29. ESI-MS: *m/z* calculated for C_18_H_20_AsNO_3_S_2_ [M]: 437.0018; [M − H]^−^: 436.0028; found 436.0057.
**TAZ2:** N-(4-(1,3,2-dithiarsinan-2-yl)phenyl)-3,4,5-trimethoxybenzamide (TA: 3,4,5-tirmethoxybenzoic acid). Yield: 234 mg (52%). ^1^H NMR (400 MHz, DMSO) δ: 10.32 (s, NH, 1H), 7.95–7.93 (d, *J* = 8 Hz, 2H), 7.83–7.81 (d, *J* = 8 Hz, 2H), 7.30 (s, 2H), 3.89 (s, 6H), 3.75 (s, 3H), 2.84–2.74 (m, 4H), 2.03–1.93 (m, 2H). ^13^C NMR (400 MHz, DMSO): 165.60, 153.11, 140.90, 140.68, 133.16, 132.48, 130.31, 129.07, 121.57, 105.85, 60.61, 56.60, 28.36, 26.15. ESI-MS: *m/z* calculated for C_19_H_22_AsNO_4_S_2_ [M]: 467.0221; [M − H]^−^: 466.0133; found 466.0178.
**TAZ5:** N-(2-(1,3,2-dithiarsinan-2-yl)phenyl)-3,4,5-trimethoxybenzamide (TA: 3,4,5-tirmethoxybenzoic acid). Yield: 131 mg (35%). ^1^H NMR (400 MHz, DMSO) δ: 10.53 (s, NH, 1H), 8.12–8.10 (d, *J* = 8 Hz, 1H), 7.55–7.51 (m, 1H), 7.45–7.41 (m, 1H), 7.38–7.36 (d, *J* = 8 Hz, 1H), 7.31 (s, 2H), 3.89 (s, 6H), 3.76 (s, 3H), 2.96–2.90 (m, 2H), 2.85–2.79 (m, 2H), 2.08–2.00 (m, 1H), 1.84–1.74 (m, 1H). ^13^C NMR (400 MHz, DMSO): 165.97, 153.14, 141.00, 140.57, 134.92, 134.47, 131.04, 129.36, 127.01, 126.33, 105.86, 60.61, 56.58, 29.19, 28.28. ESI-MS: *m/z* calculated for C_19_H_22_AsNO_4_S_2_ [M]: 467.0221; [M − H]^−^: 466.0133; found 466.0189.
**FZ2:** N-(4-(1,3,2-dithiarsinan-2-yl)phenyl)-4-fluorobenzamide (F: 4-fluorobenzoic acid). Yield: 213 mg (53%). ^1^H NMR (400 MHz, DMSO) δ: 10.46 (s, NH, 1H), 8.08–8.05 (m, 2H), 7.97–7.95 (d, *J* = 8 Hz, 2H), 7.82–7.80 (d, *J* = 8 Hz, 2H), 7.42–7.37 (m, 2H), 2.84–2.76 (m, 4H), 2.03–1.90 (m, 2H). ^13^C NMR (400 MHz, DMSO): 165.15, 163.39, 140.68, 132.52, 131.01, 129.08, 121.37, 120.86, 115.97, 115.90, 115.75, 28.35, 26.13. ESI-MS: *m/z* calculated for C_16_H_15_AsFNOS_2_ [M]: 394.9812; [M − H]^−^: 393.9722; found 393.9777.
**FZ5:** N-(2-(1,3,2-dithiarsinan-2-yl)phenyl)-4-fluorobenzamide (F: 4-fluorobenzoic acid). Yield: 124 mg (25%). ^1^H NMR (400 MHz, DMSO) δ: 10.67 (s, NH, 1H), 8.11–8.09 (d, *J* = 8 Hz, 1H), 8.05–8.02 (m, 2H), 7.54–7.50 (m, 1H), 7.44–7.37 (m, 4H), 2.94–2.87 (m, 2H), 2.82–2.76 (m, 2H), 2.04–1.97 (m, 1H), 1.84–1.75 (m, 1H). ^13^C NMR (400 MHz, DMSO): 165.58, 140.47, 131.08, 126.34, 116.03, 115.82, 29.12, 28.11. ESI-MS: *m/z* calculated for C_16_H_15_AsFNOS_2_ [M]: 394.9812; [M − H]^−^: 393.9722; found 393.9781.
**PFZ2:** N-(4-(1,3,2-dithiarsinan-2-yl)phenyl)-2,2-difluorobenzo[d][1,3]dioxole-5-carboxamide (PF:2,2-difluorobenzo[d][1,3]dioxole-5-carboxylic acid). Yield: 231 mg (25%). ^1^H NMR (400 MHz, DMSO) δ: 10.48 (s, NH, 1H), 8.01–8.00 (s, 1H), 7.96–7.94 (d, *J* = 8 Hz, 2H), 7.90–7.87 (m, 1H),7.83–7.81 (d, *J* = 8 Hz, 2H),7.62–7.60 (d, *J* = 8 Hz, 1H), 2.85–2.73 (m, 4H), 2.04–1.88 (m, 2H). ^13^C NMR (400 MHz, DMSO): 164.49, 143.19, 140.51, 133.22, 132.74, 131.92, 125.55, 121.38, 110.45, 110.23, 28.33, 26.01. ESI-MS: *m/z* calculated for C_17_H_14_AsF_2_NO_3_S_2_ [M]: 456.9621; [M − H]^−^: 455.9526; found 455.9585.
**AZ2:** 4-((4-(1,3,2-dithiarsinan-2-yl)phenyl)carbamoyl)phenyl acetate (A: 4-acetoxybenzoic acid). Yield: 44 mg (10%). ^1^H NMR (400 MHz, DMSO) δ: 10.47 (s, NH, 1H), 8.04–8.01 (d, *J* = 8 Hz, 2H), 7.98–7.96 (d, *J* = 8 Hz, 2H), 7.82–7.80 (d, *J* = 8 Hz, 2H), 7.33–7.31 (d, *J* = 8 Hz, 2H), 2.85–2.77 (m, 4H), 2.32 (s, 3H), 2.01–1.88 (m, 2H). ^13^C NMR (400 MHz, DMSO): 169.47, 165.52, 153.50, 133.19, 132.76, 129.76, 122.37, 121.32, 28.35, 26.12, 21.36. ESI-MS: *m/z* calculated for C_18_H_18_AsNO_3_S_2_ [M]: 434.9901; [M − H]^−^: 433.9871; found 433.9917; [M + Cl]^−^: 469.9638; found 469.9688.
**HDZ2:** N-(4-(1,3,2-dithiarsinan-2-yl)phenyl)-4-hydroxy-3,5-dimethoxybenzamide (HD: 4-hydroxy-3,5-dimethoxybenzoic acid). Yield: 231 mg (60%). ^1^H NMR (400 MHz, DMSO) δ: 10.50 (s, –OH, 1H), 10.29 (s, NH, 1H), 7.97–7.93 (m, 2H), 7.38–7.37 (m, 2H), 7.32 (s, 2H), 3.85 (s, 6H), 2.82–2.79 (m, 4H), 1.98–1.90 (m, 2H). ^13^C NMR (400 MHz, DMSO): 165.67, 165.55, 163.73, 152.34, 147.95, 139.77, 132.69, 129.01, 121.50, 118.13, 108.41, 107.90, 106.20, 105.24, 56.67, 28.39, 26.20, 21.23, 14.55. ESI-MS: *m/z* calculated for C_18_H_20_AsNO_4_S_2_ [M]:453.0012; [M − H]^−^: 451.9977; found 452.0033; [M + Cl]^−^: 487.9744; found 487.9800.
**MZ2:** N-(4-(1,3,2-dithiarsinan-2-yl)phenyl)-4-methylbenzamide (M: 4-methylbenzoic acid). Yield: 124 mg (35%). ^1^H NMR (400 MHz, DMSO) δ: 10.36 (s, NH, 1H), 7.98–7.96 (d, *J* = 8 Hz, 2H), 7.91–7.89 (d, *J* = 8 Hz, 2H), 7.81–7.79 (d, *J* = 8 Hz, 2H), 7.37–7.35 (d, *J* = 8 Hz, 2H), 2.85–2.74 (m, 4H), 2.51 (s, 3H), 2.04–1.89 (m, 2H). ^13^C NMR (400 MHz, DMSO): 166.06, 142.26, 140.87, 133.13, 132.32, 132.25, 129.79, 129.58, 129.42, 128.24, 121.33, 28.37, 26.15, 21.50. ESI-MS: *m/z* calculated for C_17_H_18_AsNOS_2_ [M]:391.3812; [M − H]^−^: 390.1222; found 390.1273.
**EZ2:** N-(4-(1,3,2-dithiarsinan-2-yl)phenyl)-4-ethylbenzamide (E: 4-ethylbenzoic acid). Yield: 124 mg (45%). ^1^H NMR (400 MHz, DMSO) δ: 10.36 (s, NH, 1H), 7.98–7.96 (d, *J* = 8 Hz, 2H), 7.92–7.90 (d, *J* = 8 Hz, 2H), 7.81–7.79 (d, *J* = 8 Hz, 2H), 7.40–7.38 (d, *J* = 8 Hz, 2H), 2.85–2.77 (m, 4H), 2.74–2.67 (m, 2H), 2.05–1.88 (m, 2H), 1.24–1.21 (m, 3H), ^13^C NMR (400 MHz, DMSO): 166.14, 148.41, 140.88, 133.14, 132.66, 132.24, 128.33, 128.26, 121.30, 28.56, 28.36, 26.14, 15.87. ESI-MS: *m/z* calculated for C_18_H_20_AsNOS_2_ [M]:405.0121; [M − H]^−^: 404.0133; found 404.1486.

### 4.4. Cell Culture

The HeLa and COS-7 cells were cultured in DMEM supplemented with 10% FBS and 1% penicillin/streptomycin solution. The Molm-13, NB4, and 4T1 cells were cultured in a RPMI 1640 medium under the same conditions.

### 4.5. MTT Assay

Cells were cultured in 96-well plates overnight, followed by adding 80 μL of a related medium containing compounds of different concentrations and dissolved in DMSO for 24 h or 48 h. Then, 40 μL of MTT (2.5 mg/mL) was added and the cells were incubated for another 4 h. The cells were then dissolved in 100 μL of 10% SDS solution or DMSO to release and dissolve the purple MTT products, followed by absorbance detection (BioTek, Winooski, VT, USA).

### 4.6. Molecular Docking

In order to further explore the interaction between these small molecules and the major proteins of the thioredoxin system, Ledock (http://lephar.com (accessed on 8 May 2022)) was used for docking research. The crystal structures of Trx (PDB ID: 1ERT) and TrxR (PDB ID: 3EAN) were downloaded from the Protein Data Bank (http://www.rcsb.org/pdb/home/home.do (accessed on 8 May 2022)). The process of molecular docking was carried out 50 times randomly, and the other parameters were default values. After docking, images for visualizing the types of molecular interactions were generated by Pymol software (http://www.pymol.org (accessed on 8 May 2022)).

### 4.7. Interaction between Peptide AGCUG and MAZ2

An amount of 10 μL of 2 mM MAZ2, dissolved in DMSO, was mixed with 12 μL of 2 mM AGCUG dissolved in water. The mixture was diluted to 1 mL with methanol for the ESI-MS test (Brucker Daltonics, Billerica, MA, USA), which was conducted immediately after the mix. If the incubation time had been prolonged, the reaction would have completed thoroughly and the MAZ2 content would have been less.

### 4.8. TrxR Activity

The cells planted in a 10 cm petri dish were cultured overnight, and then the compounds were added. After incubation for 12 h, the cells were collected by centrifugation and washed once with PBS. A quarter of the cells were used to measure the cellular protein content. The weight of the cell precipitation (about 0.1 g for a dish of cells) was measured, and the corresponding reagents were added according to the amount recommended by the kit instructions. Taking 0.1 g of cells as an example, 0.6 mL of reagent 1 was added to the 0.1 g of cells and homogenized in a 1 mL homogenizer in an ice bath. The solutions were broken by ultrasound for 5 min. The precipitation was centrifuged (8000× *g*, 4 °C × 10 min), and the supernatant was stored in ice-water bath for testing. The corresponding solution was added according to the kit instructions, and the absorbance at 405 nm was monitored. The measurement interval was as short as possible—10 s or 15 s—for 5 min in total. The activity was normalized to the unit protein concentration.

### 4.9. siRNA

The 4T1 cells were cultured in a DMEM medium, without serum or antibiotics, in 6-well plates for 24 h to 60% confluence, followed by incubation with siRNAs for 6 h. The transfections were performed by following the manufacturer’s instructions (TSINGKE, Beijing, China). Then, the cells were cultured in a fresh medium with serum for 24 h or 48 h prior to knockdown efficacy tests by qPCR or western blotting, respectively. The siRNA sequences are listed in Table S4.

### 4.10. PCR

See the methods in previous work [19]. The sequences are listed in Table S5.

### 4.11. ROS Level

The 4T1 cells treated with the MAZ2 of 0.8 and 1.6 µM were seeded in 6-well plates for 3, 6, 9, 12, and 24 h. Then, the medium was removed and fresh medium with DCFH-DA (0.2 µM) was added, followed by 30 min of incubation. Next, cells were collected and washed twice with PBS. Data were collected by a C6 flow cytometer (BD Biosciences, Franklin Lakes, New Jersey, USA).

The 4T1 cells treated with the MAZ2 of 0.8 and 1.6 µM were cultured in 1 cm dishes specially designed for confocal tests. Nine hours later, DCFH-DA was added for confocal tests.

The Molm-13 and 4T1 cells were cultured in 6-well plates for 6 and 12 h, followed by an MDA test according to the manufacturer’s instructions.

### 4.12. Mitochondrial Membrane Potential

The Molm-13 cells plated in 6-well plates were treated with 0.2 and 0.4 µM MAZ2 for 24 h or CCCP for 1 h. Then, cells were collected and washed twice with PBS, followed by incubation with JC-1 for 30 min and then followed by two washes with PBS. The fluorescence was monitored by a C6 flow cytometer (BD Biosciences, Franklin Lakes, NJ, USA).

### 4.13. Caspase 3 Activity

The Molm-13 cells were cultured overnight in 10 cm dishes and incubated with 0.2 µM MAZ2 for 6 h, 9h, and 12 h. The cells were collected by centrifugation (600× *g*, 4 °C × 5 min) and washed with PBS once. The cells were dissolved in 100 μL of lysate (if the lysate was not enough, the amount of lysate was increased to 150 μL or 200 μL). After 15 min, the solution was centrifuged (20,000× *g*, 4 °C × 10 min) and the supernatant was used for the following test according to the manufacturer’s instructions.

### 4.14. Apoptosis

The Molm-13 cells were cultured with 0.2, 0.3, and 0.4 µM MAZ2 for 24 h; then, the cells were collected and stained using annixin V-FITC and PE for 30 min. The separation lines were determined by single staining.

### 4.15. Mice Experiments

Animal experiments were conducted according to the guidelines of the Laboratory Animal Center of the Wuhan University College of Chemistry and Molecular Sciences. All experiments were conducted under approved procedures.

The mice (BALB/c, female, 5 weeks), purchased from Hubei Provincial Laboratory Animal Public Service Center, were injected with 0.1 mL of 4T1 cells (1 × 10^6^/mL) in the right haunch. After the tumor grew to approximately 100 mm^3^, the mice were randomly divided into three groups: control (PBS:DMSO = 8:2), 1 mg/kg, and 1.5 mg/kg. The MAZ2 was administrated once a week (i.p.) for two doses. The 1.5 mg/kg group had six mice, and the tumors of two mice disappeared after 8 days; that is, after one administration, the two tumors were almost eliminated. The body weight and tumor volume were measured every two days.

Blood samples for the blood cell content tests were collected from the eye socket veins of the mice.

### 4.16. Western Blotting

Fresh tumors and organs were minced quickly in an ice bath and homogenized in a pre-cooled Dounce Tissue Grinder (WHEATON). The homogenate was dissolved by RIPA with 0.1% PMSF. The protein concentration was determined by a BCA Protein Assay Kit (Beyotime, Shanghai, China). A total of 20–40 μg of proteins were separated by 8–12% SDS-PAGE according to the molecular weight of certain proteins, followed by being transferred onto the polyvinylidene difluoride membrane. Then, the membrane was blocked with 5% fat-free milk for 2 h, followed by incubation with primary antibodies and HRP-conjugated secondary antibodies. Then, the images were snapped by QuickChemi5100.

### 4.17. As Concentration

After being dissected, the viscera, tumors, and femurs were dried in an air-dry oven to a constant weight, which was recorded. These dry samples were dissolved in 1 mL of concentrated nitric acid and were then nitrated by heating in glass tube at 130 °C to reduce all the organic matter. Then, the remaining material was dissolved in 2 mL (0.1 mM) of nitric acid, followed by filtration through a 0.22 µm membrane filter for the ICP-MS assay. The arsenic content was normalized per gram of dry weight.

### 4.18. Pharmaceutic Kinetics

Animal experiments were conducted according to the guidelines of the Laboratory Animal Center of the Wuhan University College of Chemistry and Molecular Sciences. All experiments were conducted under approved procedure.

Female 8–10-week-old SD rats were separated into three groups (control and 1.5 mg/kg MAZ2), with three rats in every group. The MAZ2 was dissolved in a vehicle with 80% PBS and 20% DMSO. The rats were administrated intravenously once and the time started to record. Blood samples of 60 µL were collected at 0.5, 1, 2, 4, 8, 12, 24, 29, 38, and 48 h. The blood samples were dissolved by HNO_3_ nitrolysis and filtered through a 0.22 µm membrane filter. The arsenic contents were measured by ICP-MS.

### 4.19. Viscera Hematoxylin and Eosin (H & E)

The 5 organs were formalin-fixed for 24 h immediately after the mice were sacrificed, and decalcified for 3.5 h, paraffin-embedded, cut, dewaxed, and hydrated. Some samples were stained with H&E. The pictures in the results were magnified by 400× and the scale bar was 50 µm.

### 4.20. Statistics

All experiments were performed over at least two replicates. The data were presented as a mean ± SD.

## 5. Conclusions

The conjugation of 1,3-benzodioxole derivatives and their metabolites with arsenical precursors not only improved the anti-proliferation efficiency for several cancer cell lines, but also strengthened the TrxR inhibition. MAZ2 performed the best out of all the synthetic arsenicals. MAZ2 selectively inhibited cancer cell lines without influencing the normal cultured cells or the PBMC of rats, providing a wide therapeutic window. The selective inhibition of TrxR by MAZ2 caused a dramatically elevated ROS level and mitochondrial dysfunction, concomitantly leading to apoptosis.

MAZ2 reduced the Trx system protein content, especially the mitochondrial forms TrxR2 and Trx2. MAZ2 was eliminated much slower in mice and maintained an efficient concentration in the blood for a longer time than that of the arsenical precursor Z2. MAZ2 reversed the hemogram of tumor-bearing mice to normal and eliminated tumors without damage to the mice organs. The strong anti-tumor efficiency of MAZ2 with a low dosage and administration sheds light on drug development strategies and pre-clinical anti-cancer applications.

## Figures and Tables

**Table 1:** The inhibition effect of organic arsenicals in vitro ^a^.

*IC*_50_/μM	Molm		NB4		4T1		HeLa		COS-7		PBMC	
	24 h	48 h	24 h	48 h	24 h	48 h	24 h	48 h	24 h	48 h	24 h	48 h
Z2	0.60	0.53	1.50	1.20	1.67	1.89	1.01	0.70	6.1	3.2	4.6	4.0
Z5	0.45	0.41	0.40	0.30	0.87	0.84	0.30	0.25	1.75	0.81	3.5	2.8
PZ2	1.06	0.75	0.70	0.44	2.6	3.4	0.70	0.44	4.1	1.75	6.4	5.3
PZ5	0.27	0.24	0.26	0.14	0.40	0.83	0.26	0.14	1.01	0.82	5.0	4.5
PFZ2	0.80	0.70	0.50	0.30	1.00	0.80	0.50	0.15	6.0	3.5	6.3	5.7
HDZ2	0.60	0.60	0.80	0.50	1.00	1.01	2.0	1.01	9.1	3.5	5.8	4.9
TAZ2	1.60	1.50	1.00	0.60	3.5	2.2	2.0	1.04	9.1	5.2	7.3	6.5
TAZ5	0.71	0.70	0.20	0.15	0.70	0.70	0.80	0.50	2.0	1.80	2.2	1.54
DAZ2	0.90	0.75	1.71	1.14	1.50	1.20	2.3	1.06	7.1	3.3	6.2	5.7
DAZ5	0.45	0.32	0.80	0.51	0.60	0.50	0.81	0.55	1.61	1.27	4.1	3.5
MAZ2	0.20	0.10	0.40	0.30	0.80	0.40	0.40	0.30	5.2	3.3	7.6	6.9
FZ2	1.20	1.00	0.40	0.30	2.3	2.2	1.20	0.40	3.5	2.2	5.5	4.7
FZ5	0.50	0.50	0.15	0.15	0.70	0.60	0.35	0.15	1.10	0.80	2.4	1.82
MZ2	1.20	0.80	0.40	0.30	1.20	0.91	0.61	0.40	3.4	1.21	3.4	2.7
EZ2	1.10	1.02	0.41	0.30	1.40	1.40	1.01	0.81	4.2	2.1	3.7	2.9
AZ2	1.10	1.00	0.60	0.60	2.0	1.00	0.80	0.50	2.2	2.1	5.2	4.9

^a^ The data (*IC*_50_) are expressed as the means of at least three independent experiments and the unit is µmol·L^−1^. The data (*IC*_50_) are expressed as the mean ± SD in Tables S1 and S2.

## Data Availability

The data presented in this study are available in Supplementary Materials.

## Supplementary Materials

The following supporting information can be downloaded at: https://www.mdpi.com/article/10.3390/ijms23136930/s1.
