# Supplementary material for: 1,3-Benzodioxole Derivatives Improve the Anti-Tumor Efficiency of Arsenicals
Source: Int J Mol Sci. 2022 Jun 22;23(13):6930. doi: 10.3390/ijms23136930 (PMC9266561; doi:10.3390/ijms23136930)
Supplement: Supplementary file 1 [file ijms-23-06930-s001.zip › ijms-1740508-supplementary.pdf]

International Journal of
Molecular Sciences

MDPI

Article: Supporting Information

1,3-Benzodioxole Derivatives Improve the Anti-Tumor Efficiency of Arsenicals

Xue-Min Shi [1], Wen-Yan She [1], Ting-Ting Liu [2], Lian-Xun Gao [1], Yu-Jiao Liu [2,*] and Yi Liu [1,2,*]

[1] College of Chemistry and Molecular Science, Wuhan University, Wuhan 430072, China; xueminshichem@whu.edu.cn (X.-M.S.); wenyanshe@whu.edu.cn (W.-Y.S.); lxgaochem@whu.edu.cn (L.-X.G.)

[2] State Key Laboratory of Separation Membranes and Membrane Processes, School of Chemistry, Tiangong University, Tianjin 300387, China; 2120020069@tiangong.edu.cn

* Correspondence: yujiaoliu@tiangong.edu.cn or yujiaoliu@whu.edu.cn (Y.-J.L.); yiliuchem@whu.edu.cn (Y.L.); Tel.: +86-27-68753465 (Y.L.); Fax.: +86-27-68754067 (Y.L.)

contents

Characterization of chemical structure 2

MTT assay of arsenicals to 5 cell lines... 27

MAZ2 inhibited TrxR activity by binding to the C-terminal Sec/Cys pair 28

MAZ2 administration had no obvious side effects... 31

siRNA 31

PCR.. 31

Characterization of chemical structure

Z2

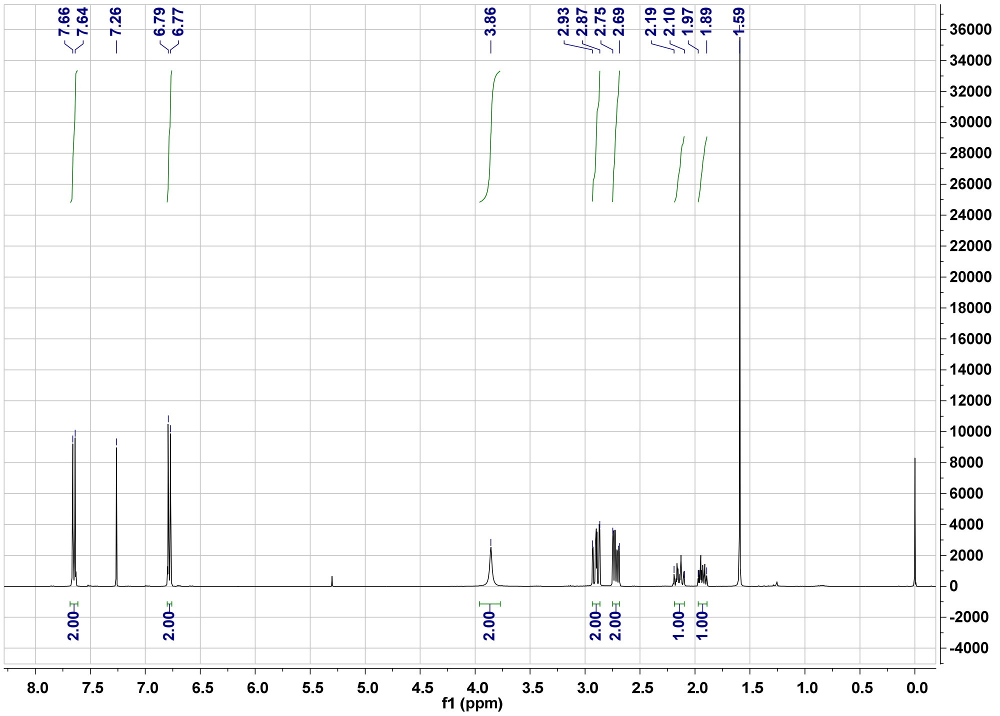

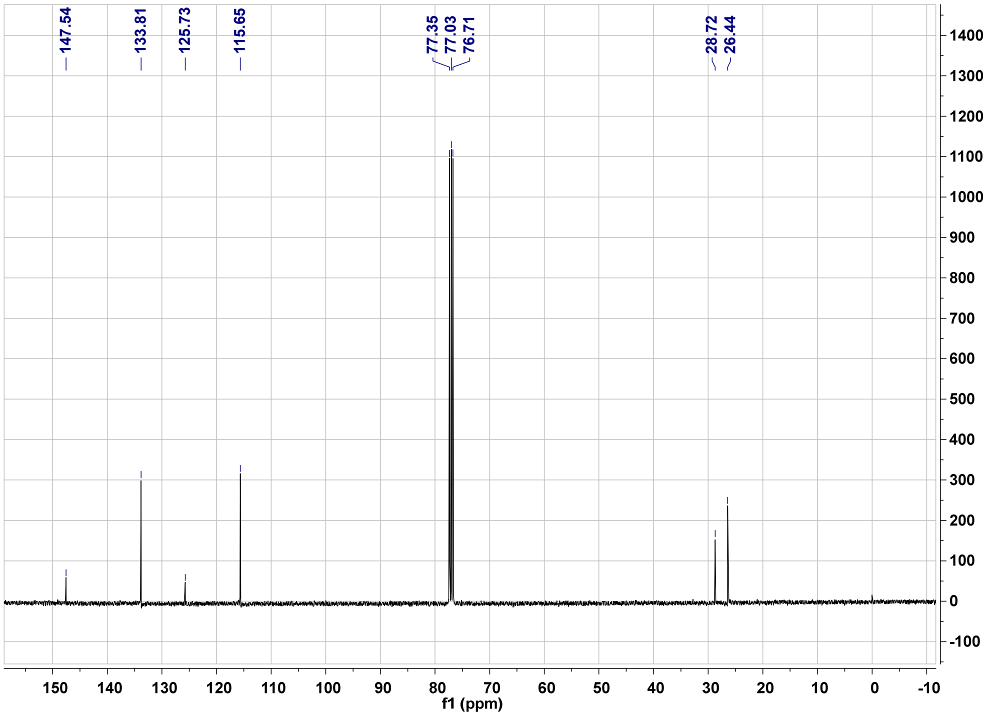

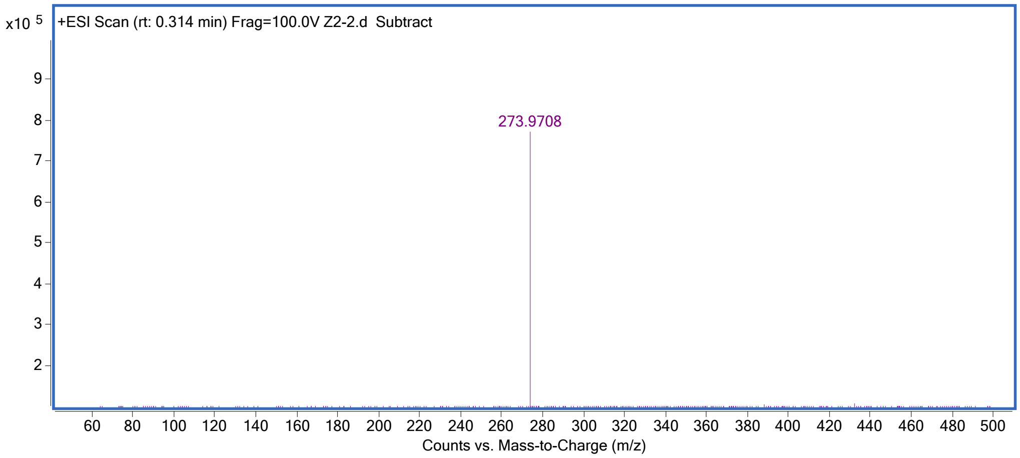
x10 5
+ESI Scan (rt: 0.314 min) Frag=100.0V Z2-2.d Subtract
273.9708
Counts vs. Mass-to-Charge (m/z)

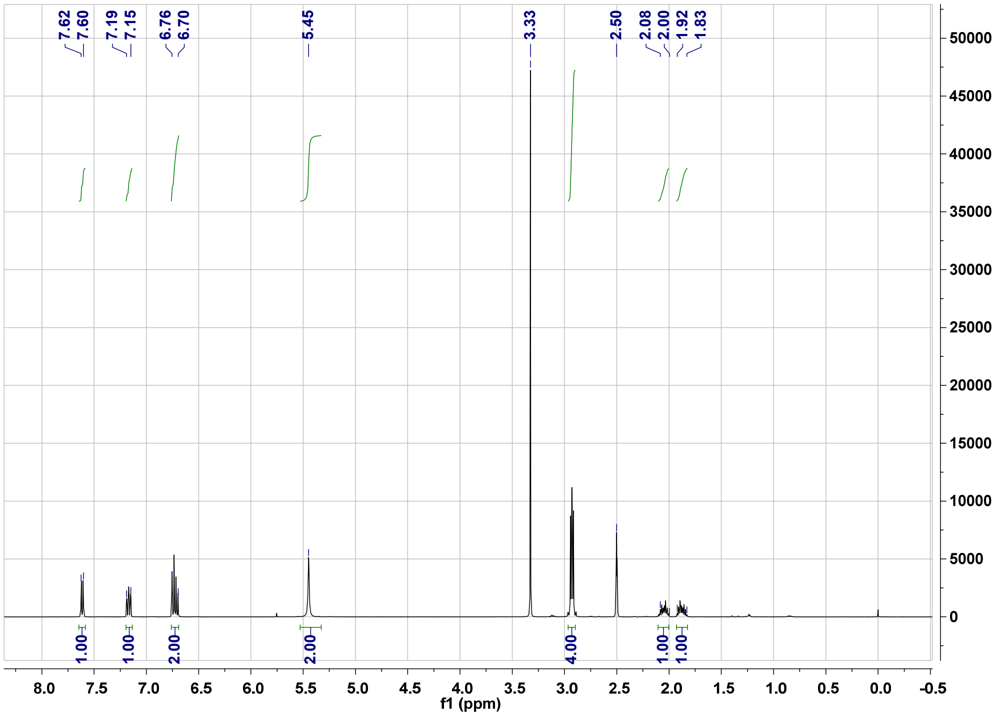
7.62
7.60
7.19
7.15
6.76
6.70
5.45
3.33
2.50
2.08
2.00
1.92
1.83
1.00
1.00
2.00
2.00
4.00
1.00
1.00
f1 (ppm)

Z5

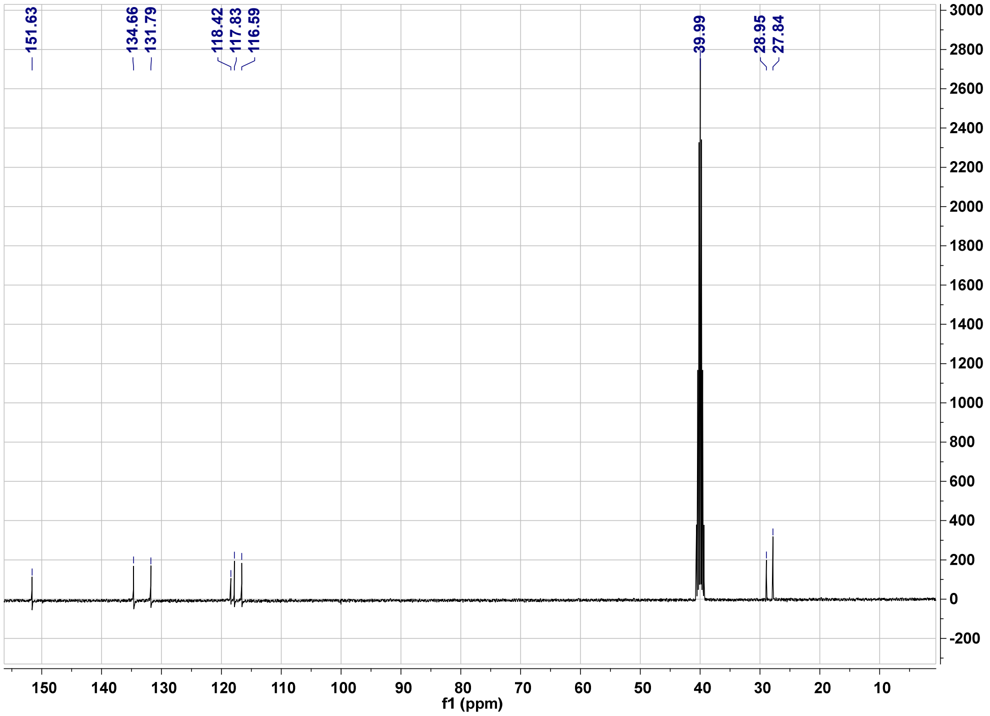
151.63
134.66
131.79
118.42
117.83
116.59
39.99
28.95
27.84
f1 (ppm)

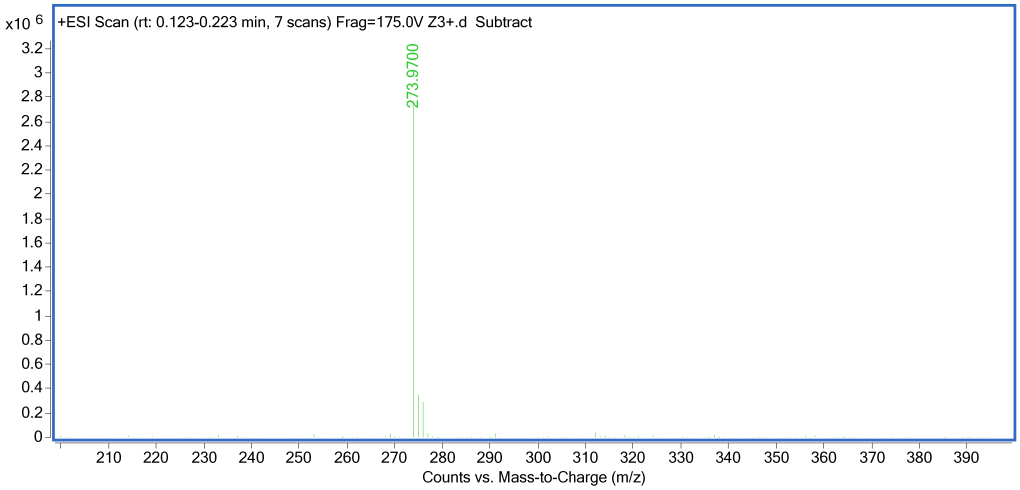
+ESI Scan (rt: 0.123-0.223 min, 7 scans) Frag=175.0V Z3+.d Subtract
273.9700
Counts vs. Mass-to-Charge (m/z)

PZ2

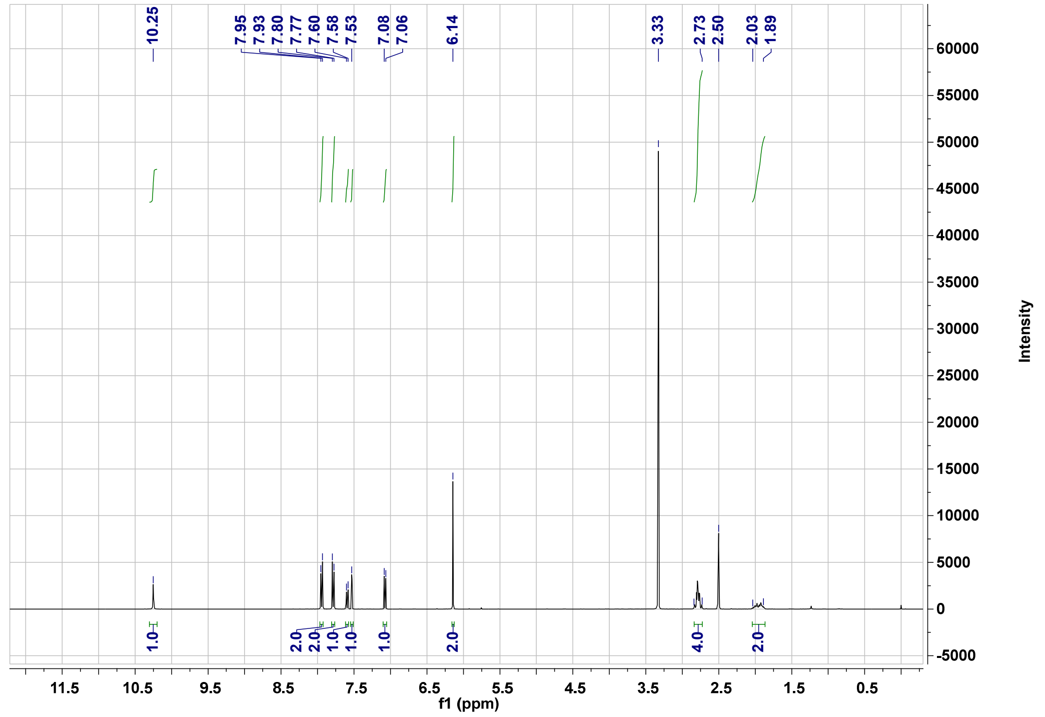
10.25
7.95
7.93
7.80
7.77
7.60
7.58
7.53
7.08
7.06
6.14
3.33
2.73
2.50
2.03
1.89
1.0
2.0
2.0
1.0
1.0
1.0
2.0
4.0
2.0
f1 (ppm)
Intensity

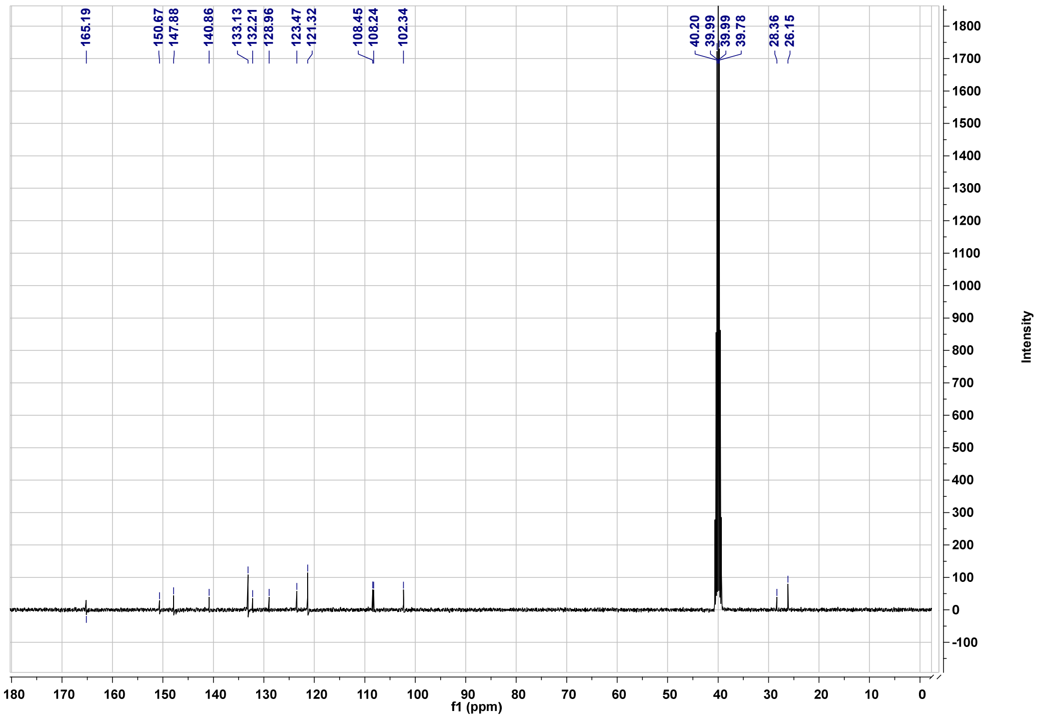
165.19
150.67
147.88
140.86
133.13
132.21
128.96
123.47
121.32
108.45
108.24
102.34
40.20
39.99
39.99
39.78
28.36
26.15
f1 (ppm)
Intensity

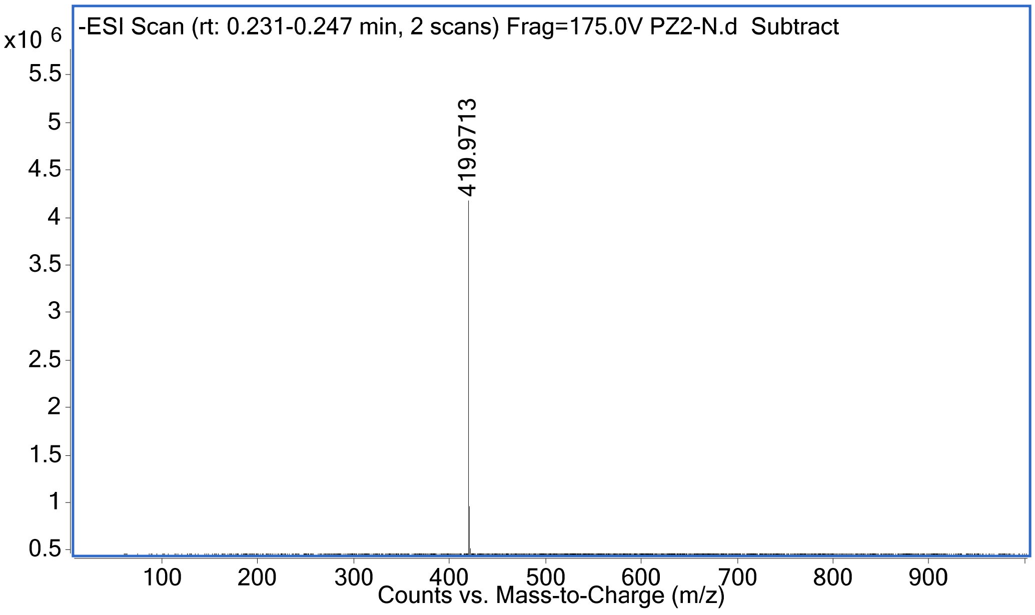
-ESI Scan (rt: 0.231-0.247 min, 2 scans) Frag=175.0V PZ2-N.d Subtract
x10 6
419.9713
Counts vs. Mass-to-Charge (m/z)

PZ5

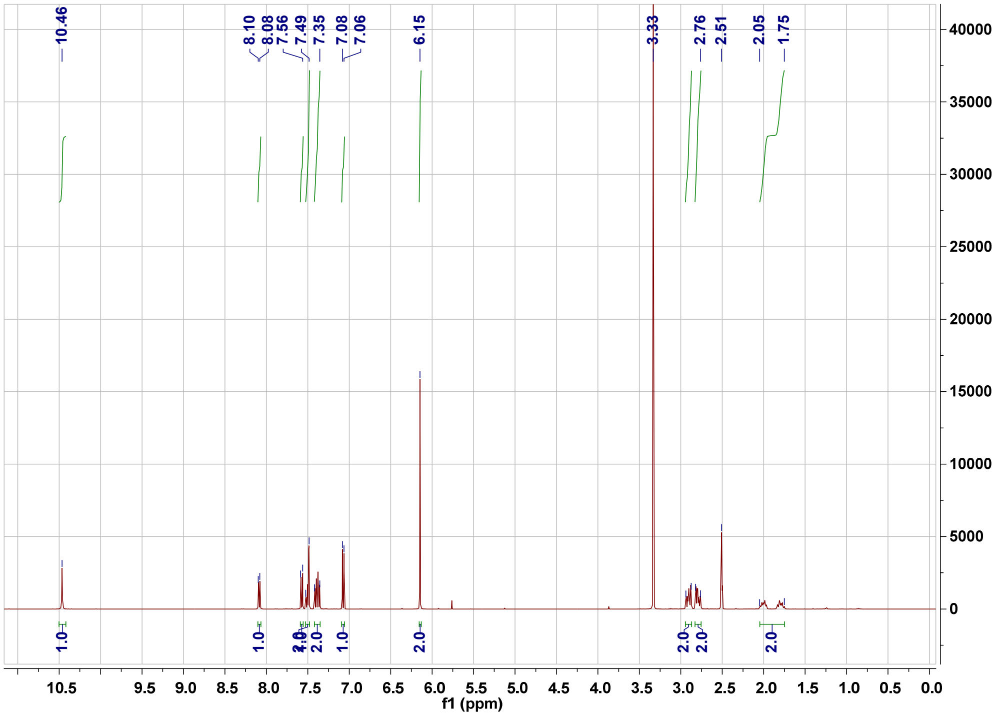
f1 (ppm)

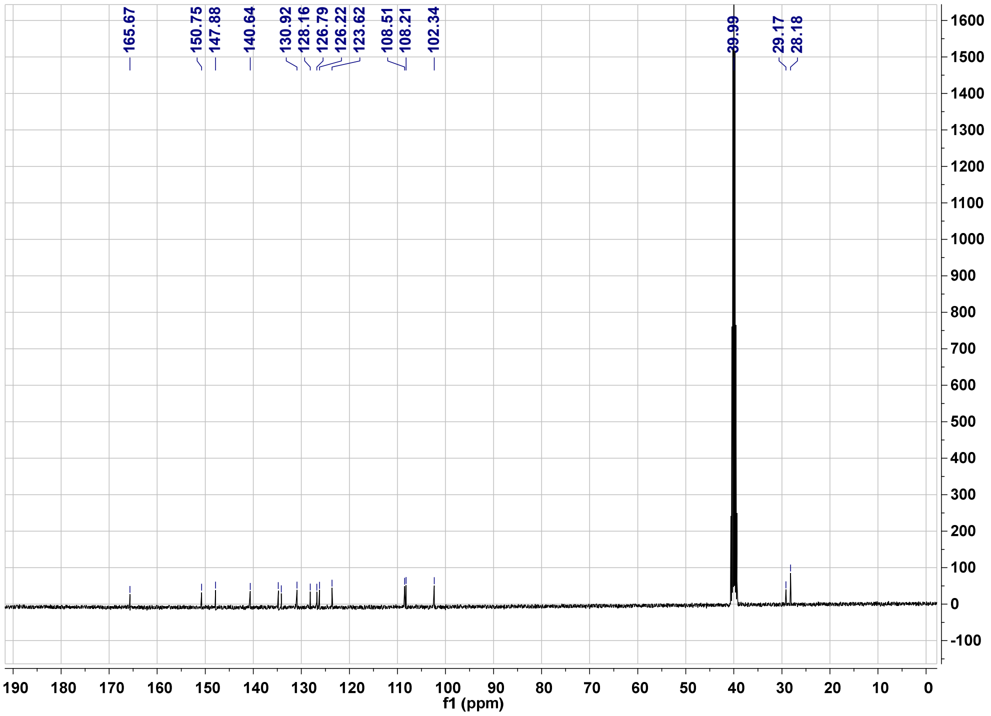
165.67
150.75
147.88
140.64
130.92
128.16
126.79
126.22
123.62
108.51
108.21
102.34
39.99
29.17
28.18
f1 (ppm)

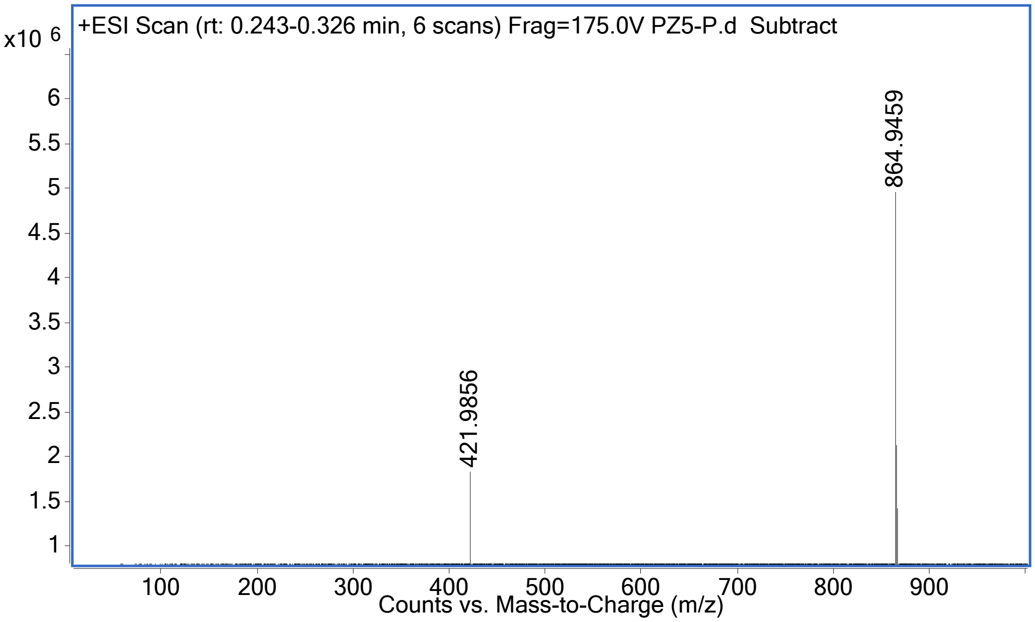
+ESI Scan (rt: 0.243-0.326 min, 6 scans) Frag=175.0V PZ5-P.d Subtract
x10 6
864.9459
421.9856
Counts vs. Mass-to-Charge (m/z)

PFZ2

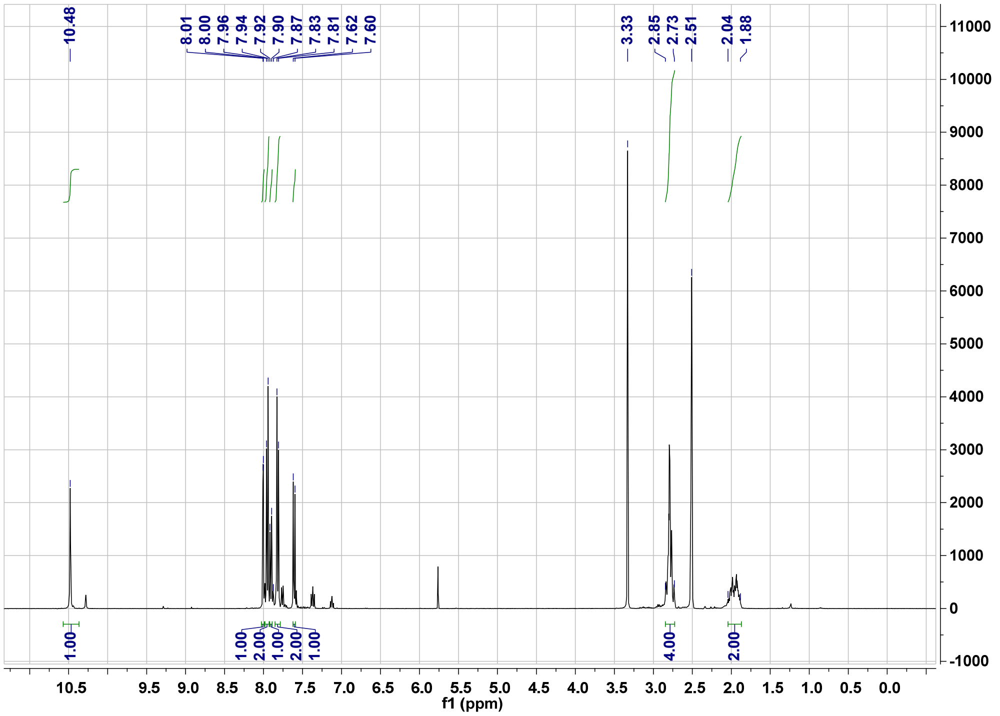
10.48
8.01
8.00
7.96
7.94
7.92
7.90
7.87
7.83
7.81
7.62
7.60
3.33
2.85
2.73
2.51
2.04
1.88
1.00
2.00
1.00
2.00
1.00
4.00
2.00
f1 (ppm)

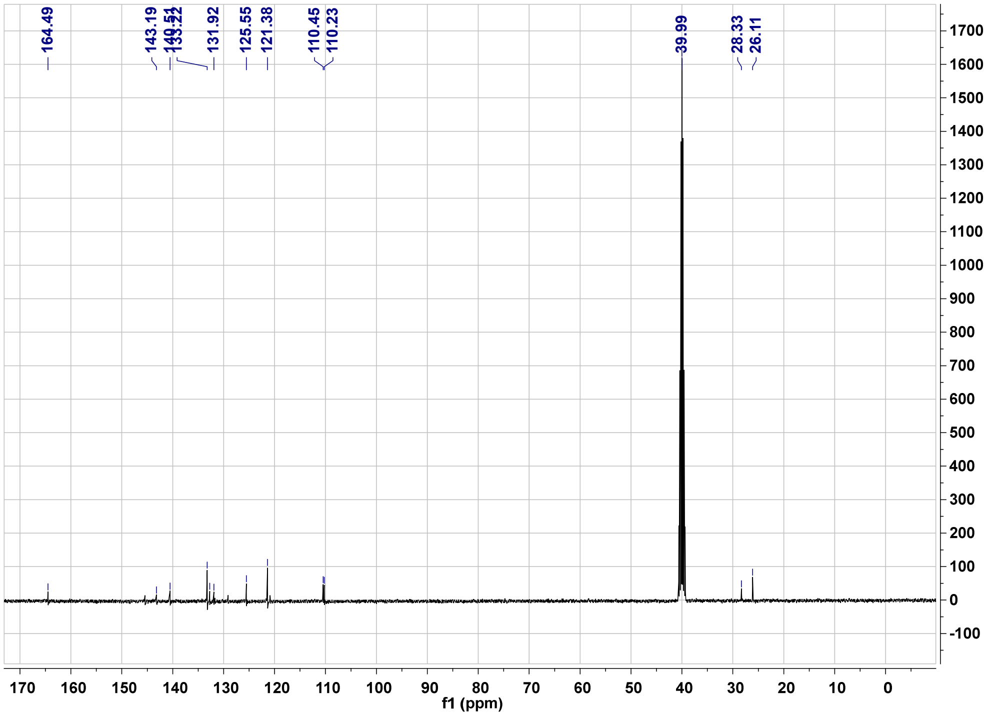
164.49
143.19
131.92
125.55
121.38
110.45
110.23
39.99
28.33
26.11
f1 (ppm)

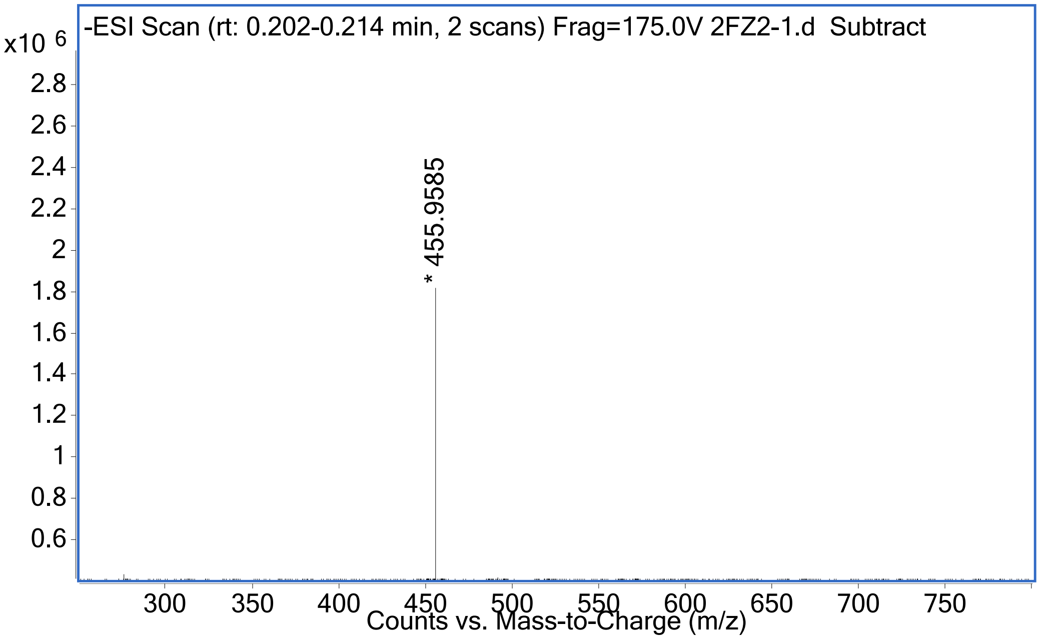
-ESI Scan (rt: 0.202-0.214 min, 2 scans) Frag=175.0V 2FZ2-1.d Subtract
x10 6
* 455.9585
Counts vs. Mass-to-Charge (m/z)

HDZ2

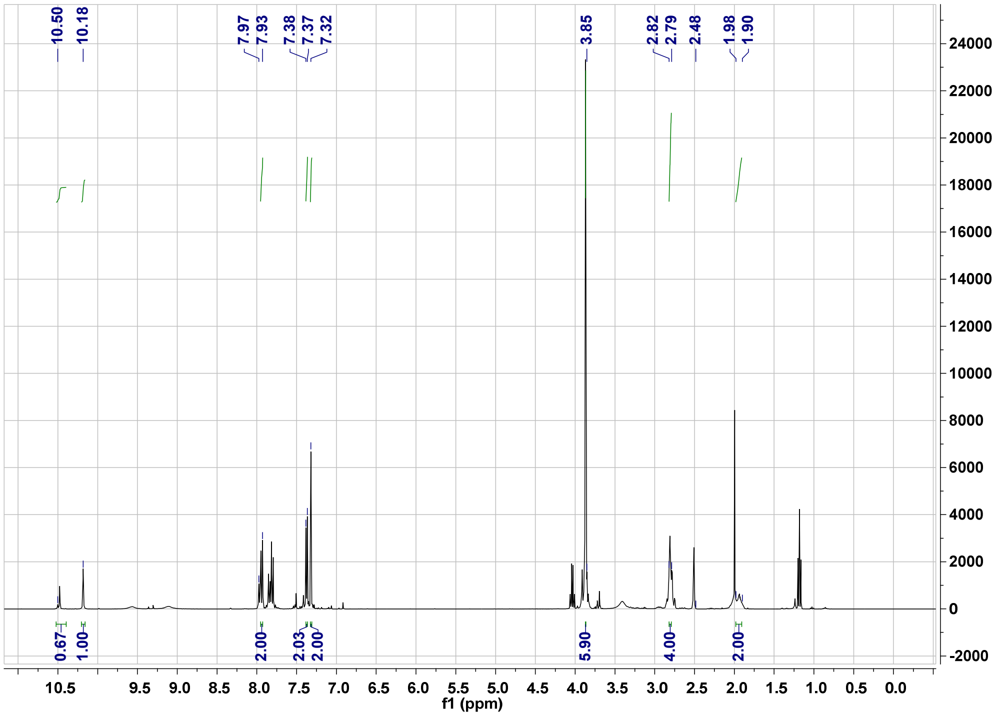
10.50
10.18
7.97
7.93
7.38
7.37
7.32
3.85
2.82
2.79
2.48
1.98
1.90
0.67
1.00
2.00
2.03
2.00
5.90
4.00
2.00
f1 (ppm)

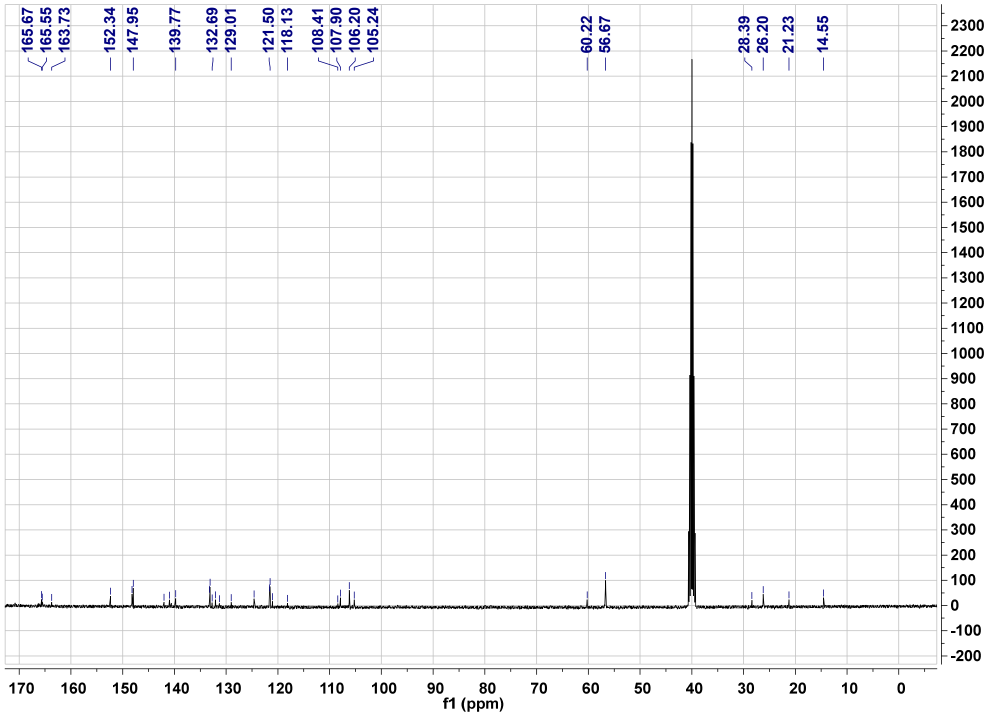
165.67
165.55
163.73
152.34
147.95
139.77
132.69
129.01
121.50
118.13
108.41
107.90
106.20
105.24
60.22
56.67
28.39
26.20
21.23
14.55
f1 (ppm)

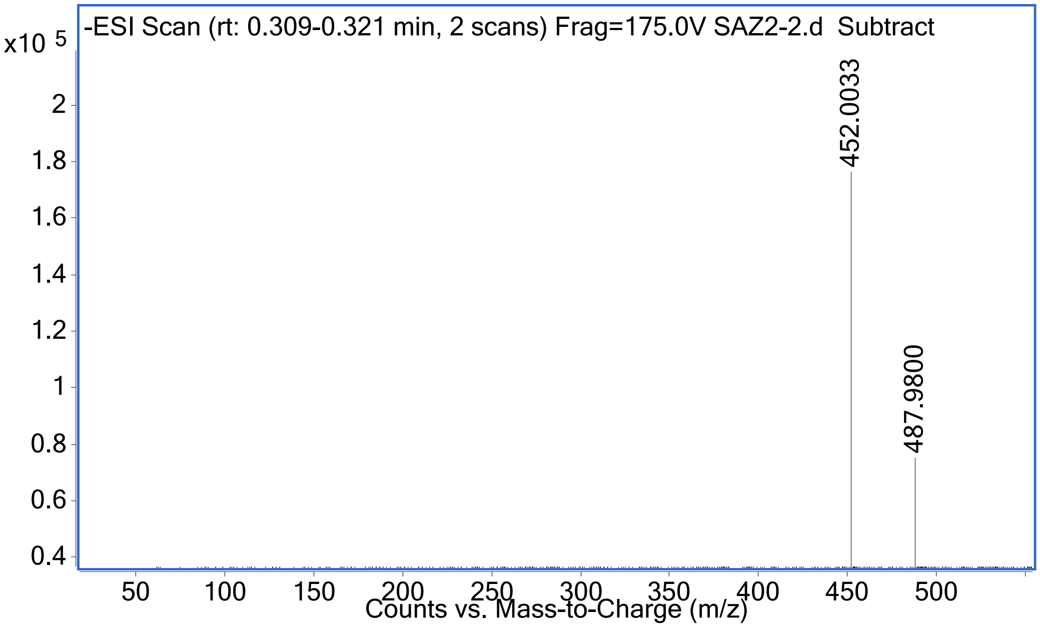
-ESI Scan (rt: 0.309-0.321 min, 2 scans) Frag=175.0V SAZ2-2.d Subtract
x10 5
452.0033
487.9800
Counts vs. Mass-to-Charge (m/z)

TAZ2

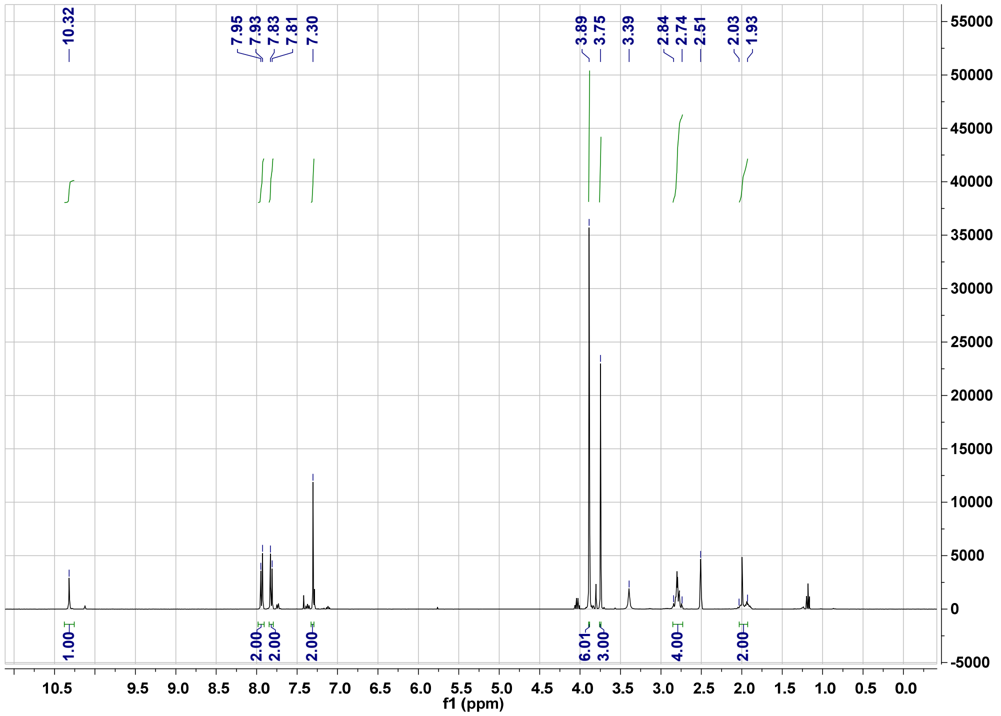
10.32
7.95
7.93
7.83
7.81
7.30
3.89
3.75
3.39
2.84
2.74
2.51
2.03
1.93
1.00
2.00
2.00
2.00
6.01
3.00
4.00
2.00
f1 (ppm)

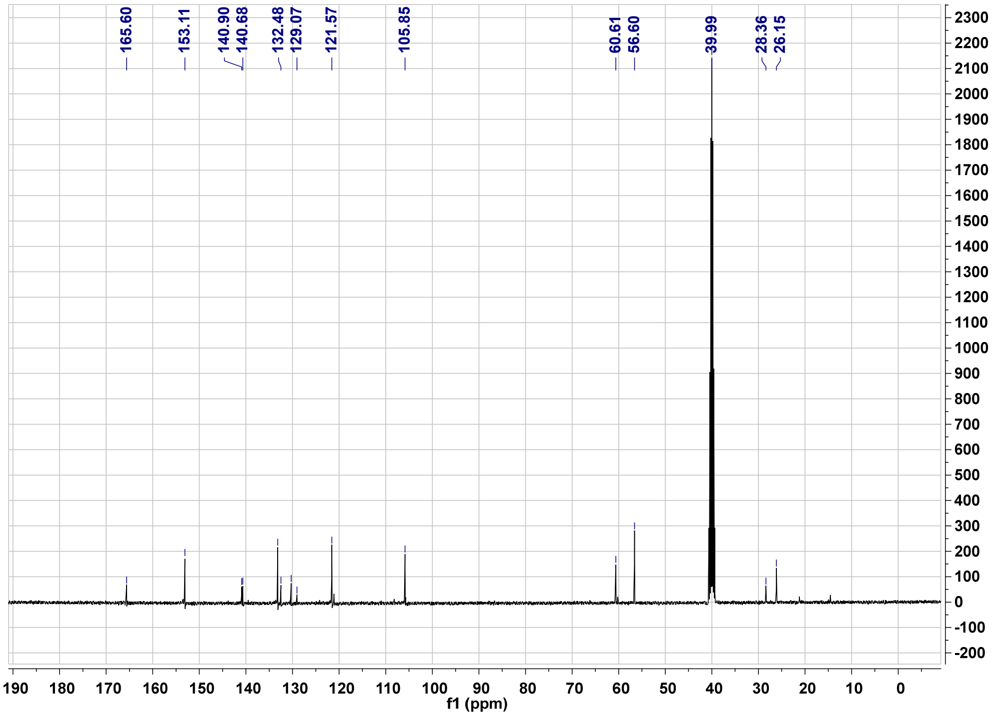
165.60
153.11
140.90
140.68
132.48
129.07
121.57
105.85
60.61
56.60
39.99
28.36
26.15
f1 (ppm)

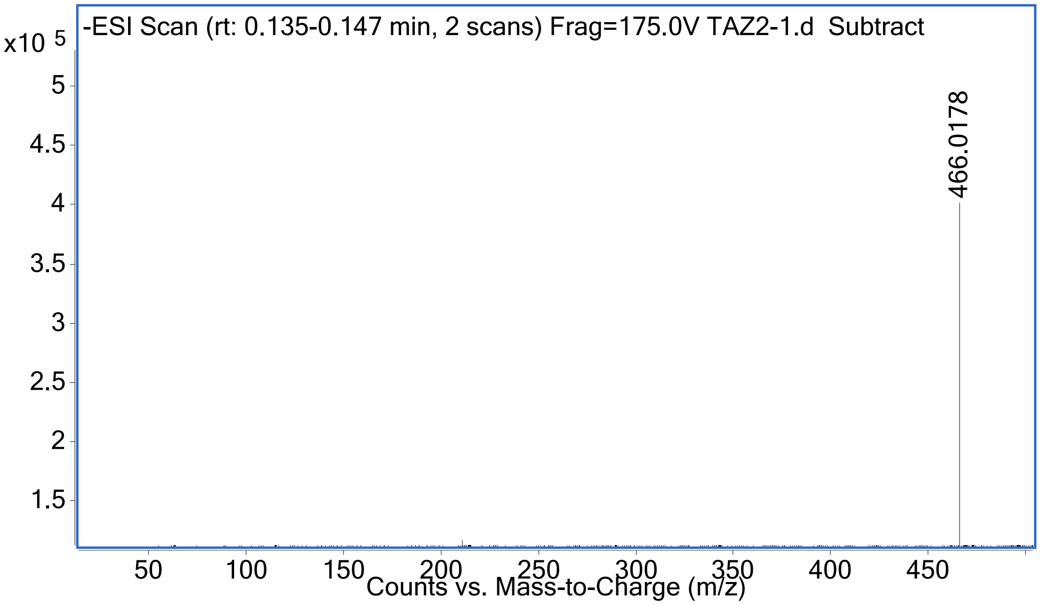
-ESI Scan (rt: 0.135-0.147 min, 2 scans) Frag=175.0V TAZ2-1.d Subtract
x10 5
466.0178
Counts vs. Mass-to-Charge (m/z)

TAZ5

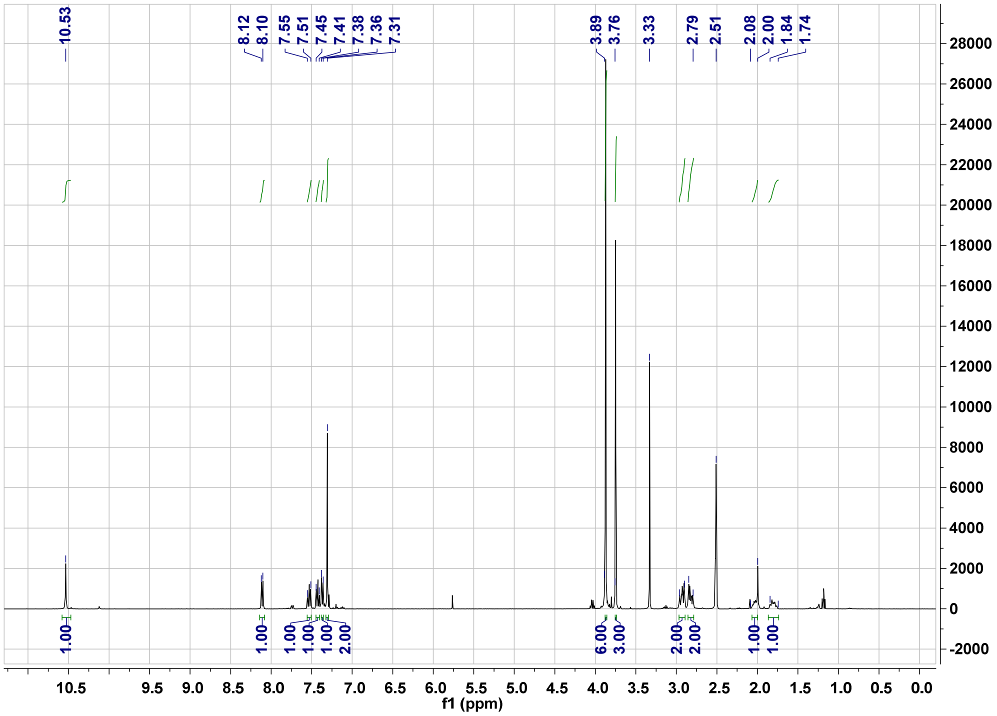
10.53
8.12
8.10
7.55
7.51
7.45
7.41
7.38
7.36
7.31
3.89
3.76
3.33
2.79
2.51
2.08
2.00
1.84
1.74
f1 (ppm)

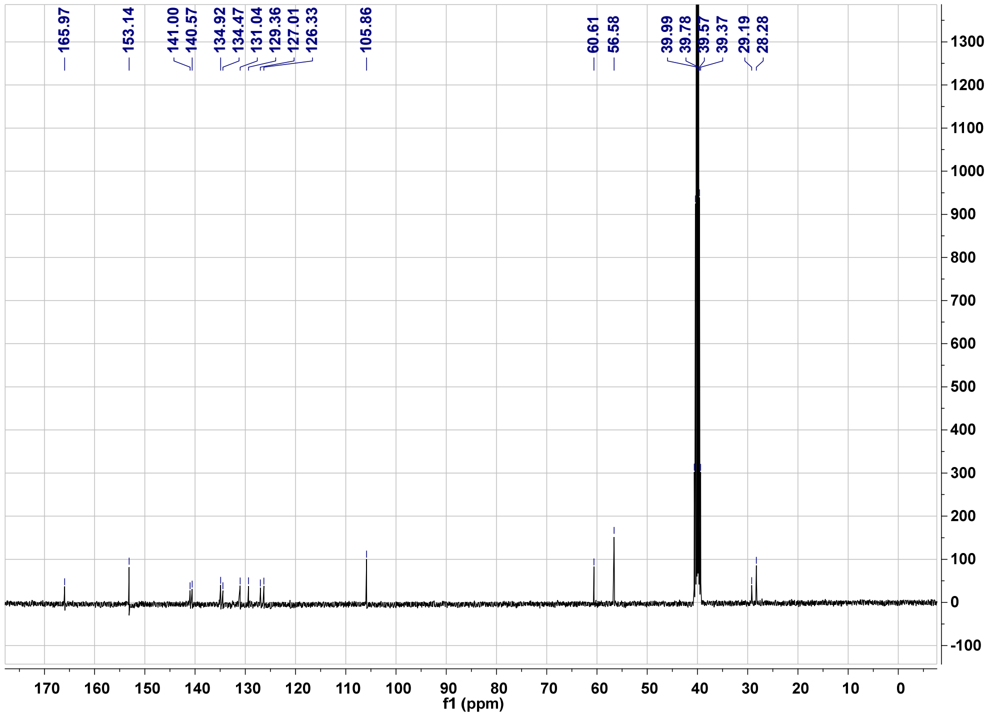
165.97
153.14
141.00
140.57
134.92
134.47
131.04
129.36
127.01
126.33
105.86
60.61
56.58
39.99
39.78
39.57
39.37
29.19
28.28
f1 (ppm)

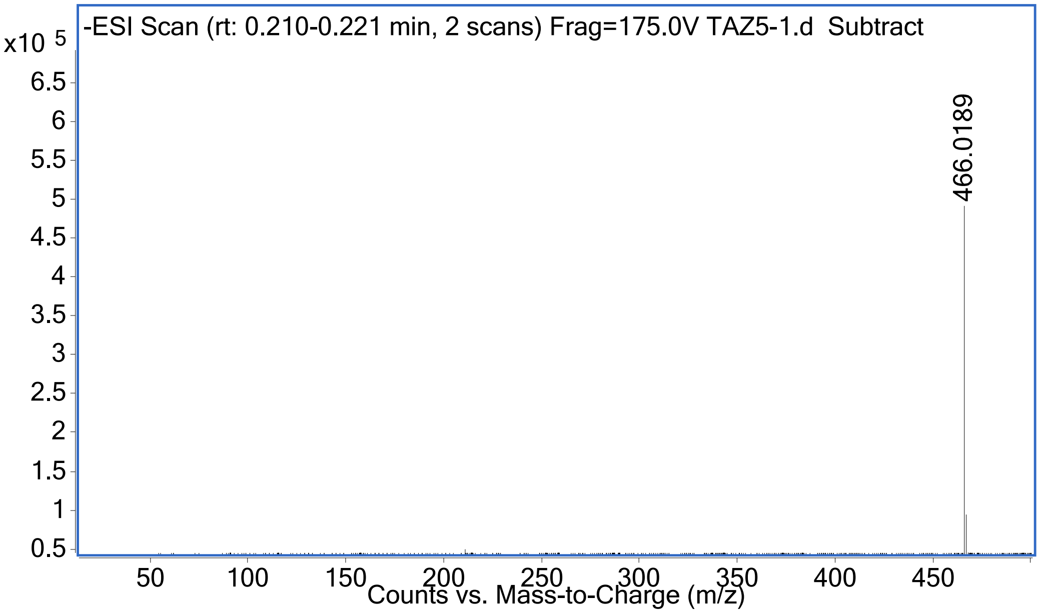
-ESI Scan (rt: 0.210-0.221 min, 2 scans) Frag=175.0V TAZ5-1.d Subtract
x10 5
466.0189
Counts vs. Mass-to-Charge (m/z)

DAZ2

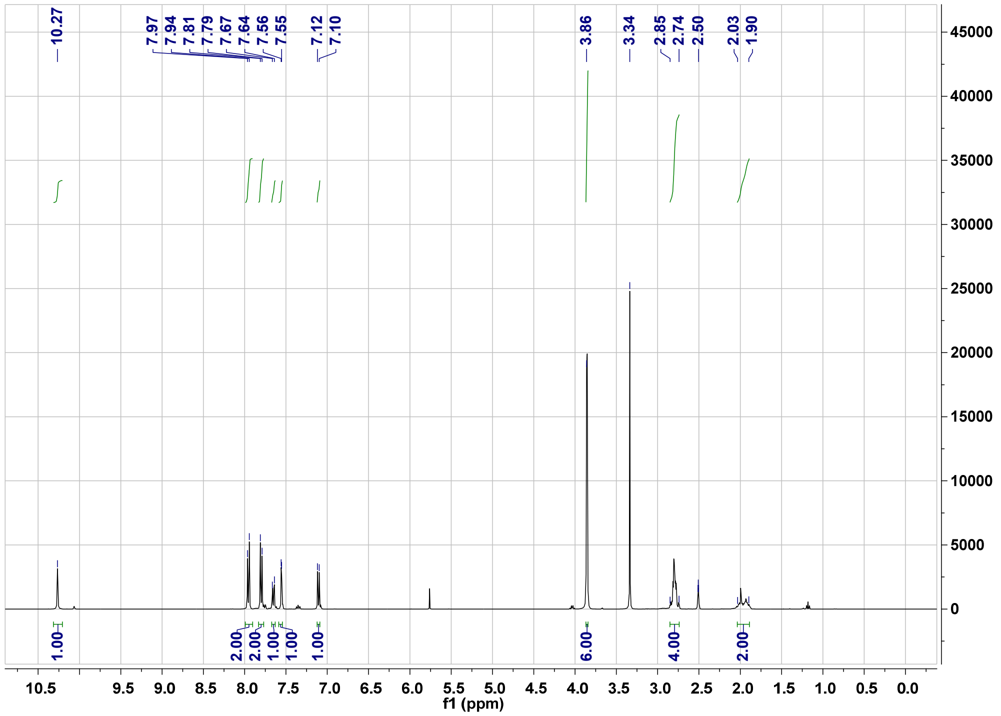
10.27
7.97
7.94
7.81
7.79
7.67
7.64
7.56
7.55
7.12
7.10
3.86
3.34
2.85
2.74
2.50
2.03
1.90
1.00
2.00
2.00
1.00
1.00
1.00
6.00
4.00
2.00
f1 (ppm)

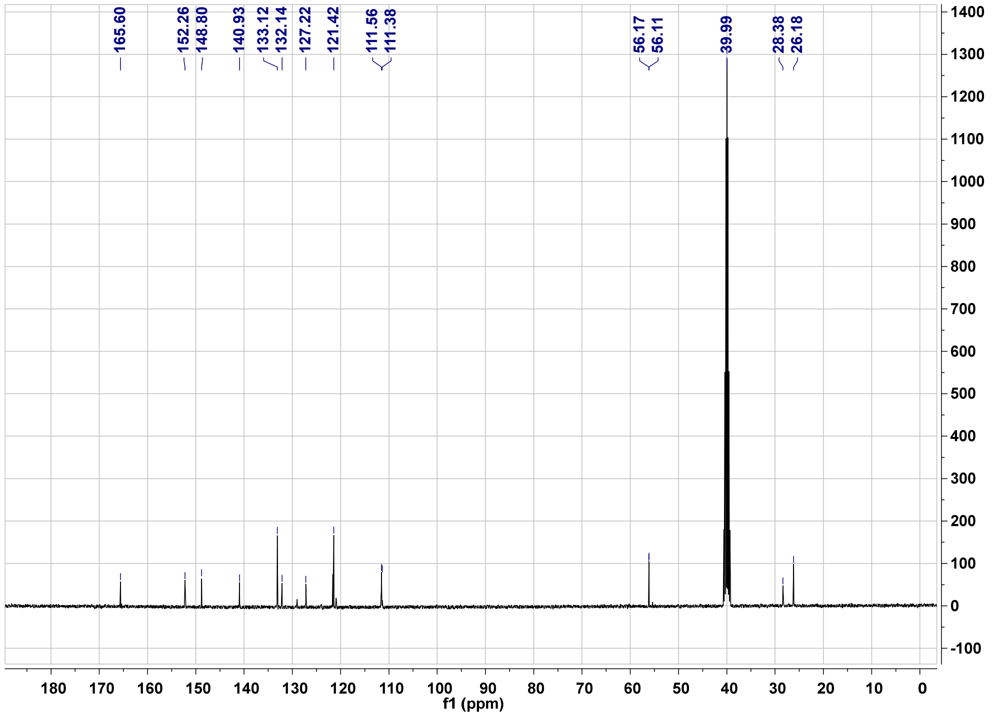
165.60
152.26
148.80
140.93
133.12
132.14
127.22
121.42
111.56
111.38
56.17
56.11
39.99
28.38
26.18
f1 (ppm)

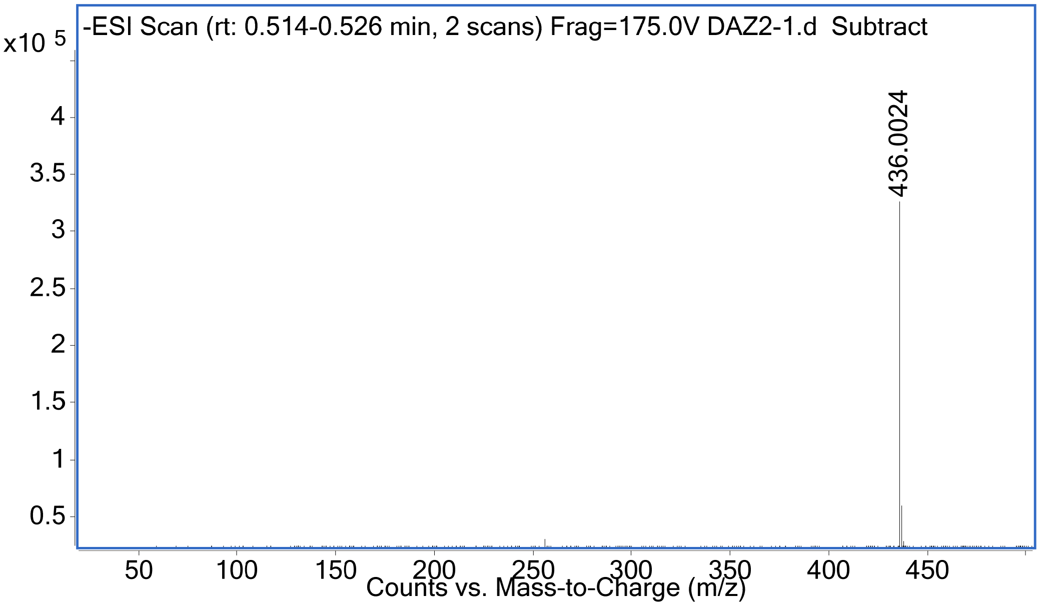
x10 5
-ESI Scan (rt: 0.514-0.526 min, 2 scans) Frag=175.0V DAZ2-1.d Subtract
436.0024
Counts vs. Mass-to-Charge (m/z)

DAZ5

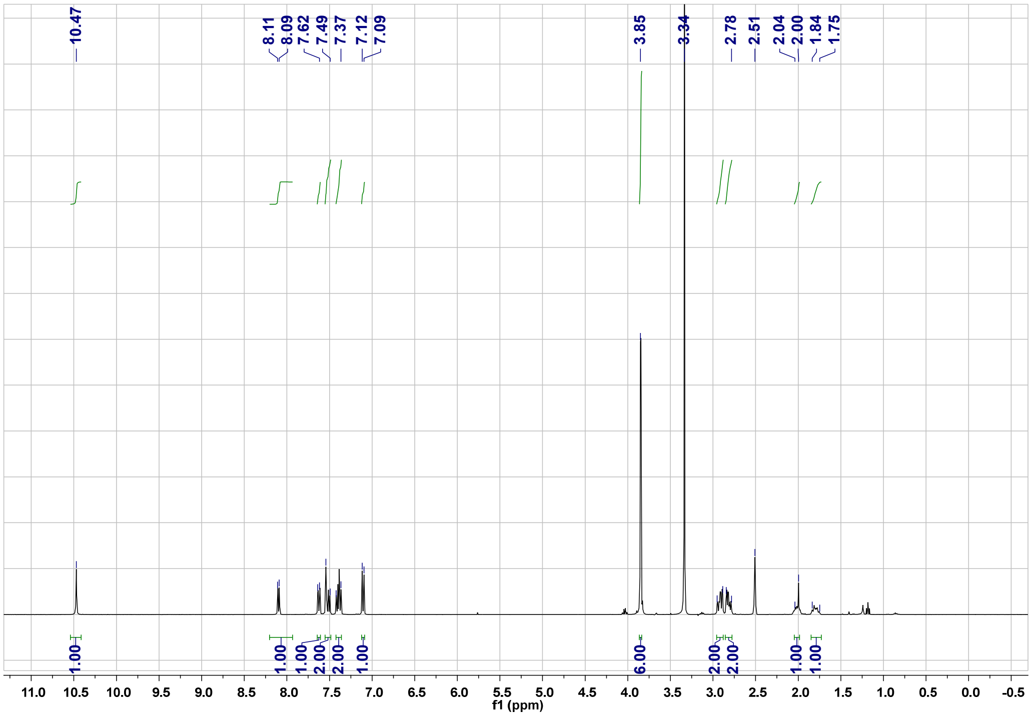
10.47
8.11
8.09
7.62
7.49
7.37
7.12
7.09
3.85
3.34
2.78
2.51
2.04
2.00
1.84
1.75
f1 (ppm)

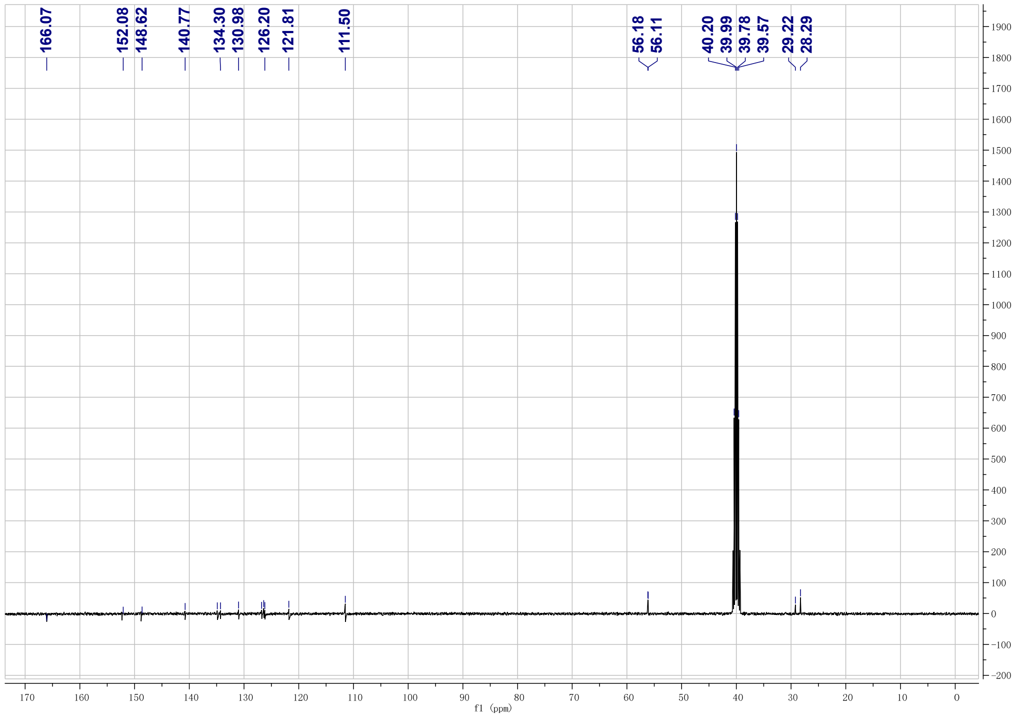
166.07
152.08
148.62
140.77
134.30
130.98
126.20
121.81
111.50
56.18
56.11
40.20
39.99
39.78
39.57
29.22
28.29
f1 (ppm)

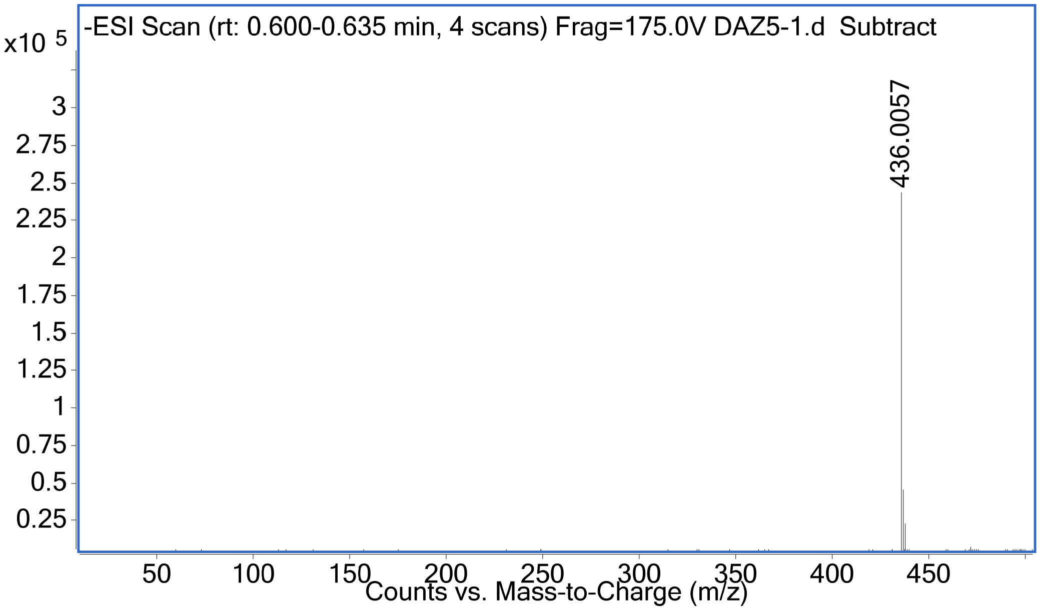
-ESI Scan (rt: 0.600-0.635 min, 4 scans) Frag=175.0V DAZ5-1.d Subtract
x10 5
436.0057
Counts vs. Mass-to-Charge (m/z)

MAZ2

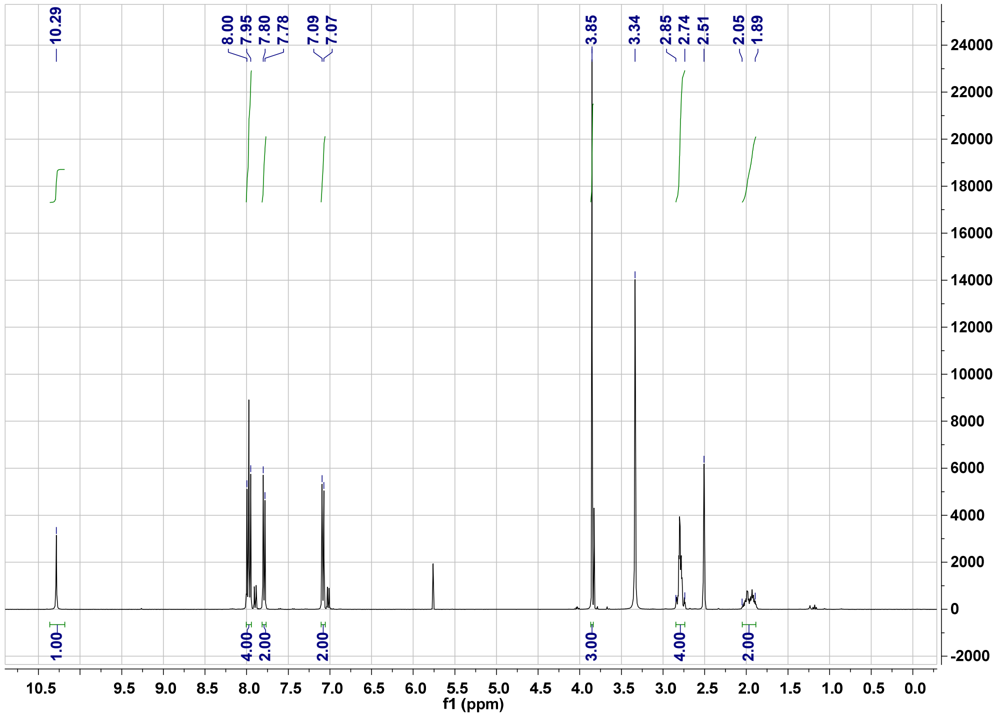
10.29
8.00
7.95
7.80
7.78
7.09
7.07
3.85
3.34
2.85
2.74
2.51
2.05
1.89
1.00
4.00
2.00
2.00
3.00
4.00
2.00
f1 (ppm)

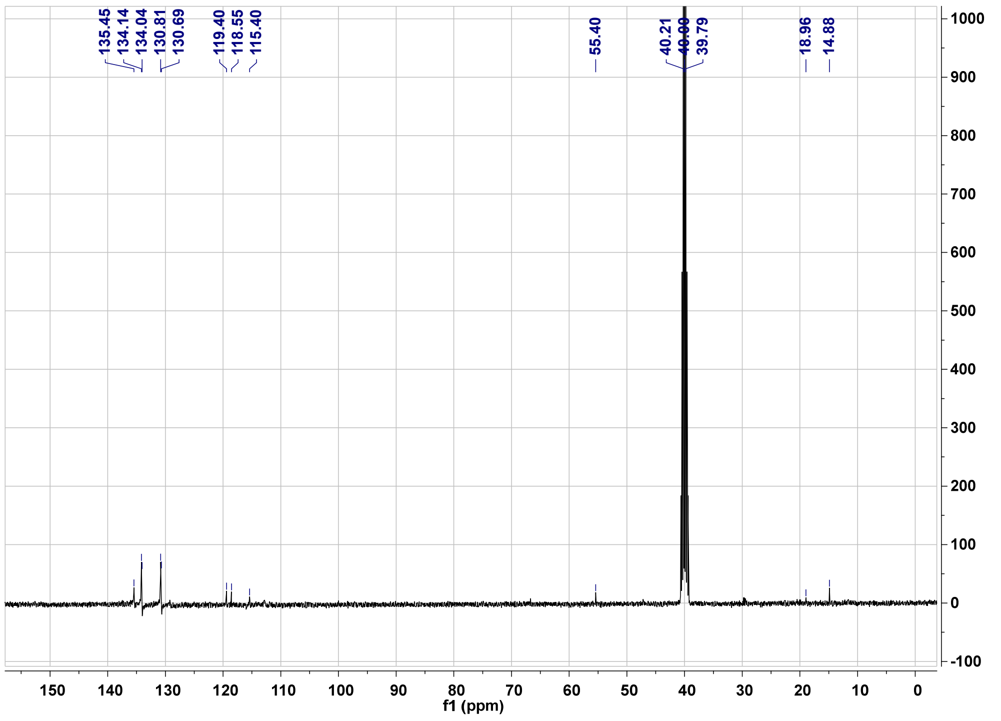
135.45
134.14
134.04
130.81
130.69
119.40
118.55
115.40
55.40
40.21
39.79
18.96
14.88
f1 (ppm)

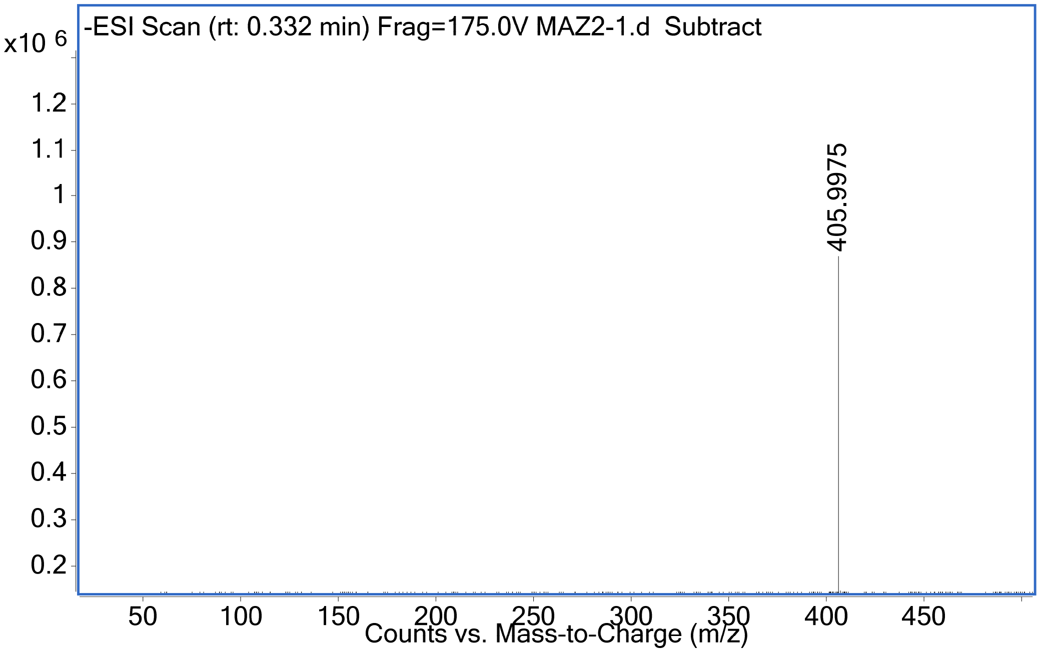
-ESI Scan (rt: 0.332 min) Frag=175.0V MAZ2-1.d Subtract
x10 6
1.2
1.1
1
0.9
0.8
0.7
0.6
0.5
0.4
0.3
0.2
405.9975
50
100
150
200
250
300
350
400
450
Counts vs. Mass-to-Charge (m/z)

FZ2

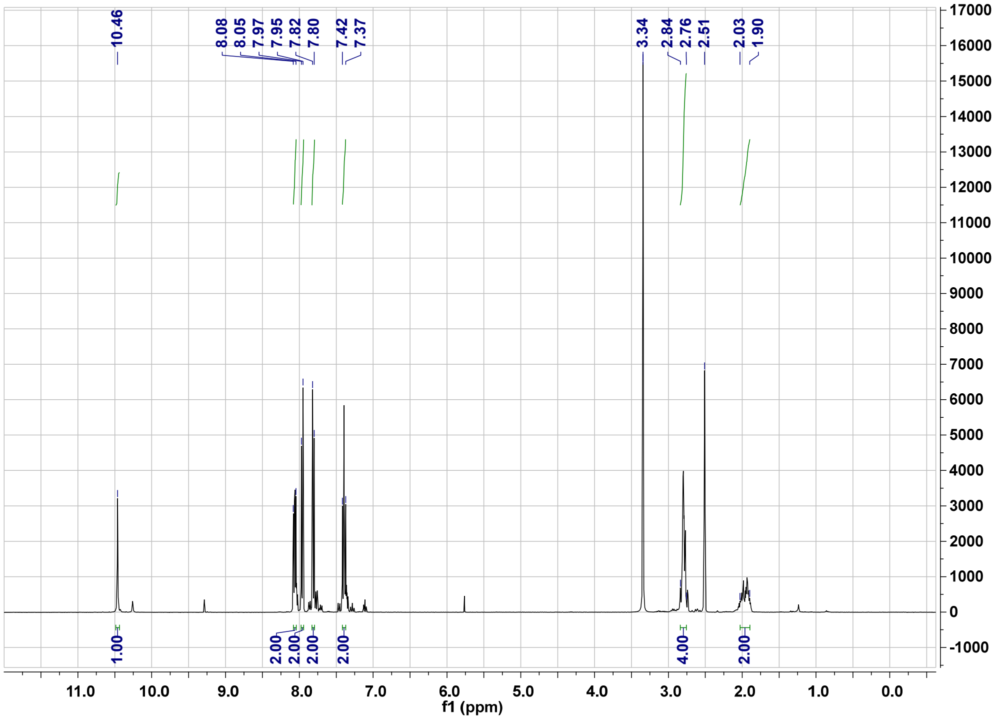
10.46
8.08
8.05
7.97
7.95
7.82
7.80
7.42
7.37
3.34
2.84
2.76
2.51
2.03
1.90
1.00
2.00
2.00
2.00
2.00
4.00
2.00
f1 (ppm)

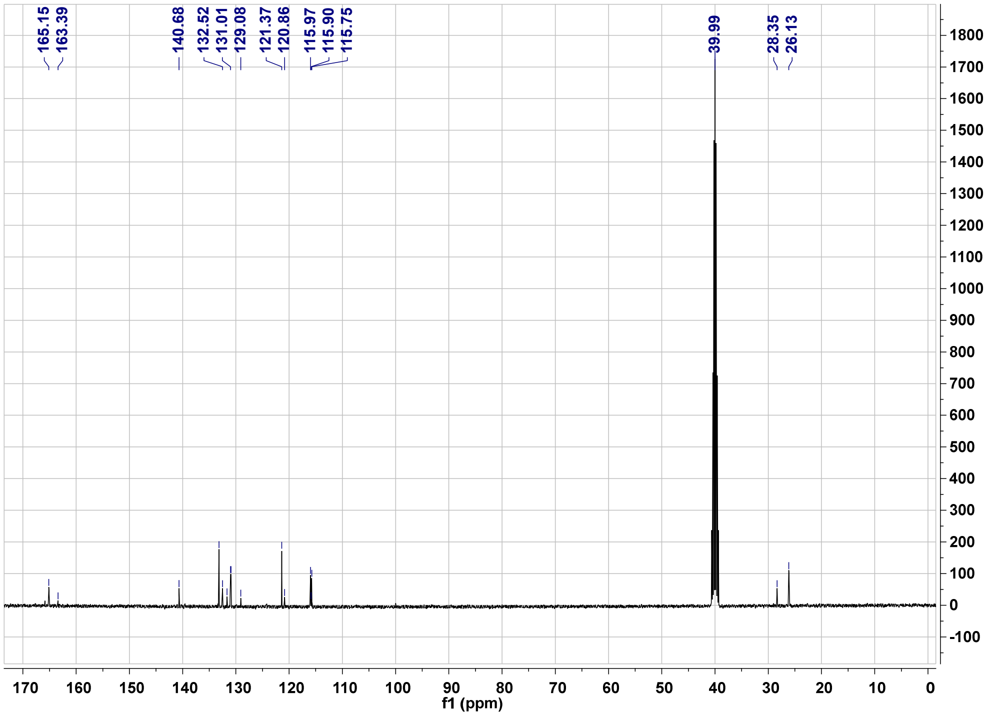
165.15
163.39
140.68
132.52
131.01
129.08
121.37
120.86
115.97
115.90
115.75
39.99
28.35
26.13
f1 (ppm)

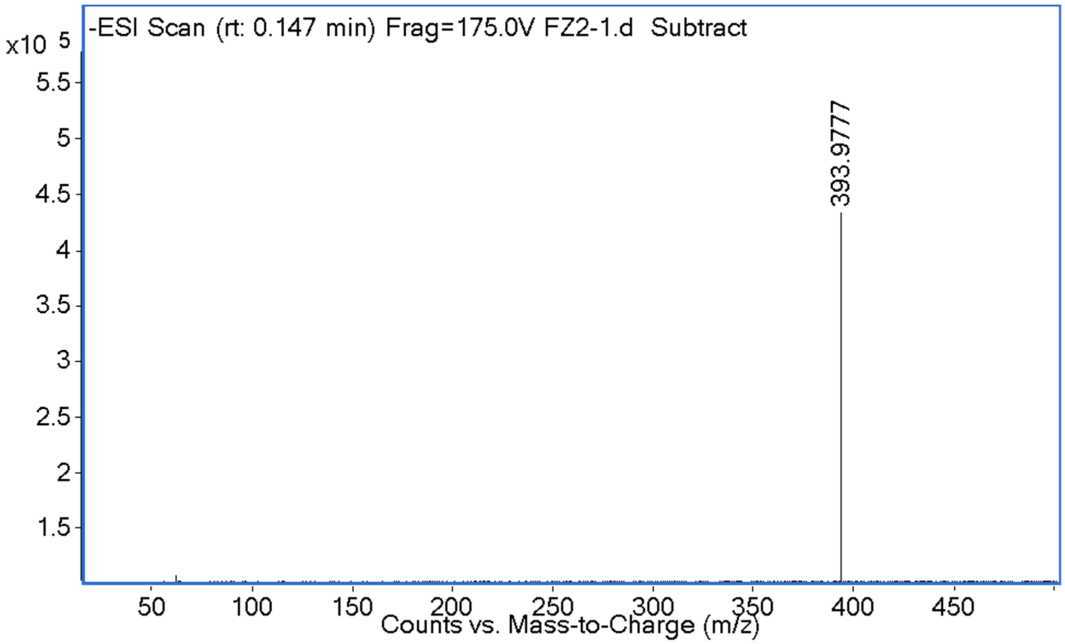
-ESI Scan (rt: 0.147 min) Frag=175.0V FZ2-1.d Subtract
x10 5
393.9777
Counts vs. Mass-to-Charge (m/z)

FZ5

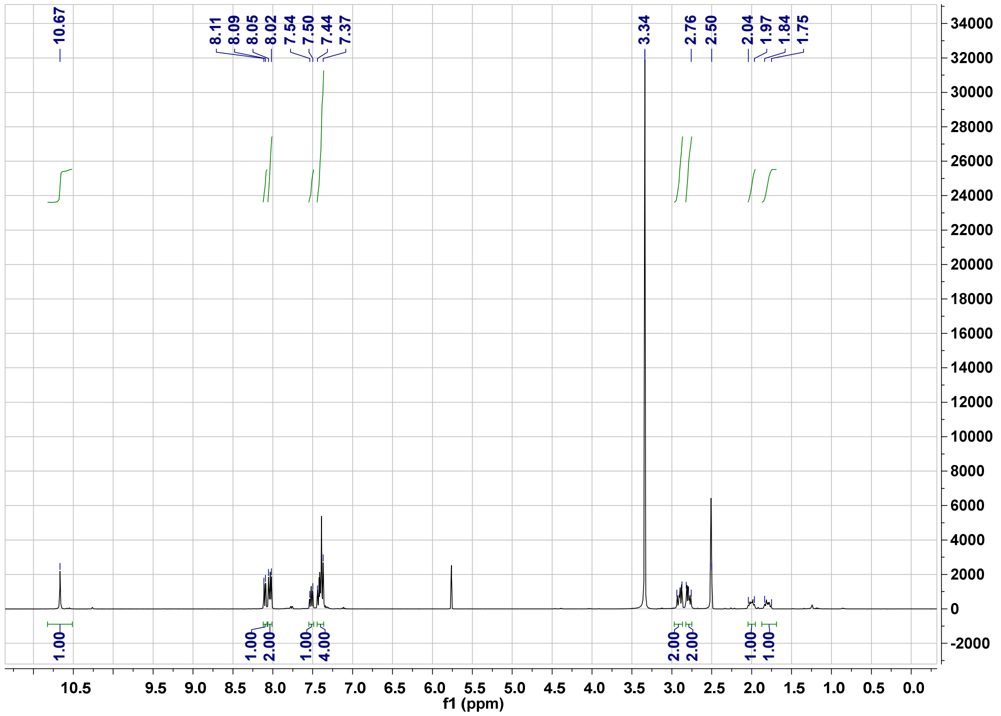
10.67
8.11
8.09
8.05
8.02
7.54
7.50
7.44
7.37
3.34
2.76
2.50
2.04
1.97
1.84
1.75
1.00
1.00
2.00
1.00
4.00
2.00
2.00
1.00
1.00
f1 (ppm)

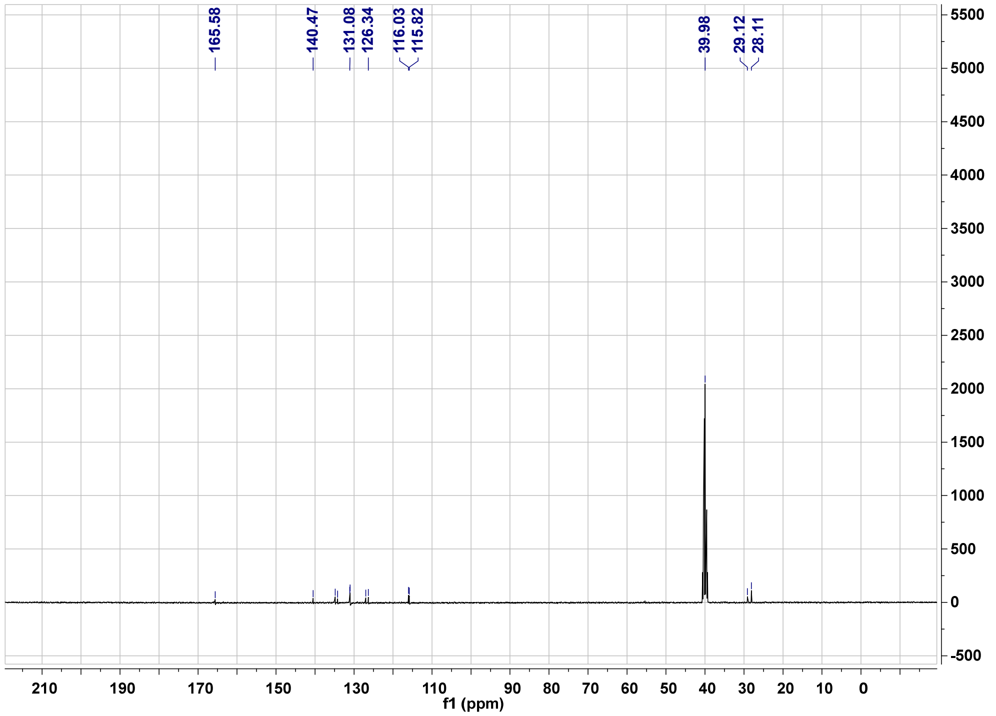
165.58
140.47
131.08
126.34
116.03
115.82
39.98
29.12
28.11
f1 (ppm)

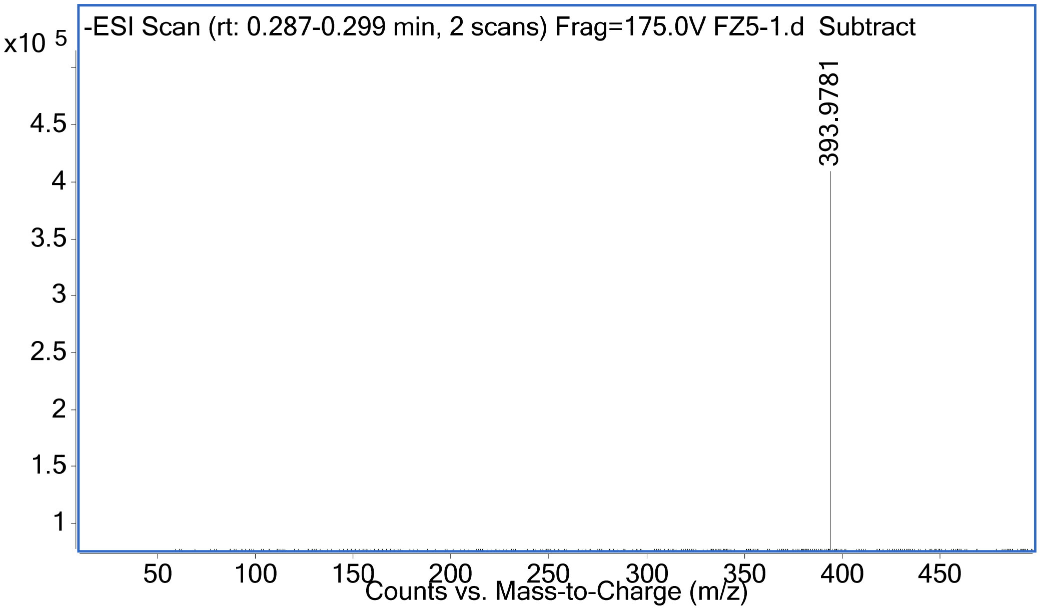
-ESI Scan (rt: 0.287-0.299 min, 2 scans) Frag=175.0V FZ5-1.d Subtract
x10 5
393.9781
Counts vs. Mass-to-Charge (m/z)

MZ2

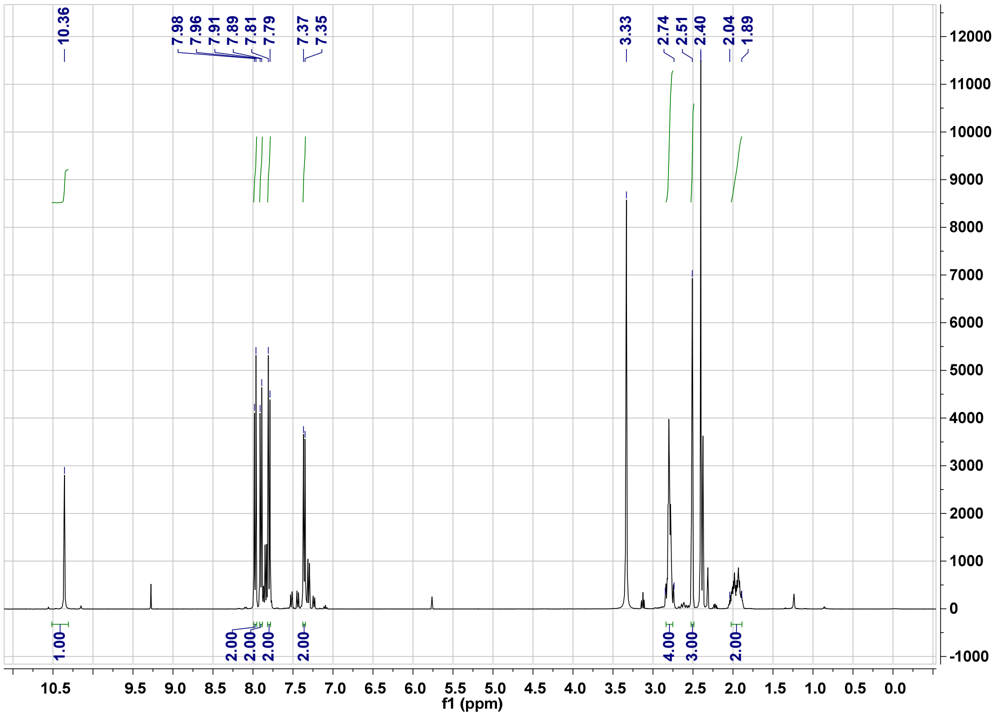
10.36
7.98
7.96
7.91
7.89
7.81
7.79
7.37
7.35
3.33
2.74
2.51
2.40
2.04
1.89
1.00
2.00
2.00
2.00
2.00
4.00
3.00
2.00
f1 (ppm)

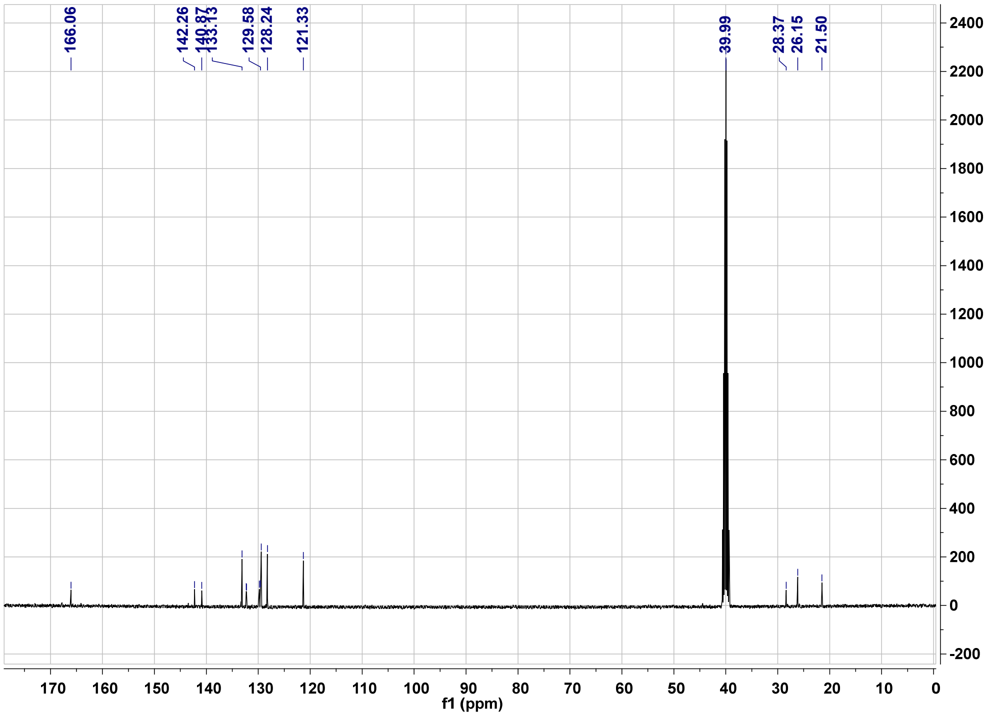
166.06
142.26
133.13
129.58
128.24
121.33
39.99
28.37
26.15
21.50
f1 (ppm)

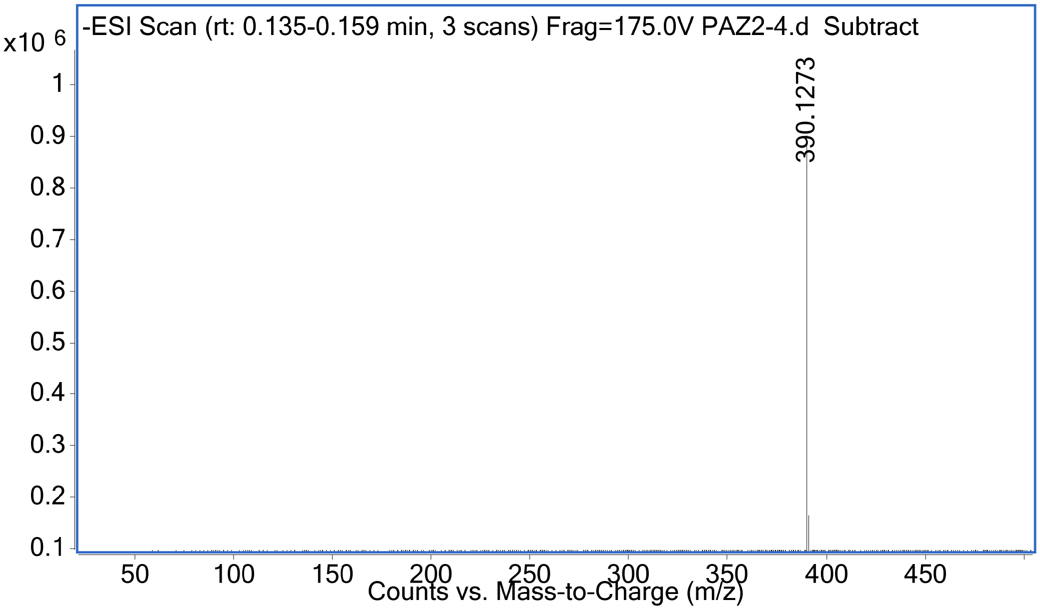
-ESI Scan (rt: 0.135-0.159 min, 3 scans) Frag=175.0V PAZ2-4.d Subtract
x10 6
390.1273
Counts vs. Mass-to-Charge (m/z)

EZ2-003191

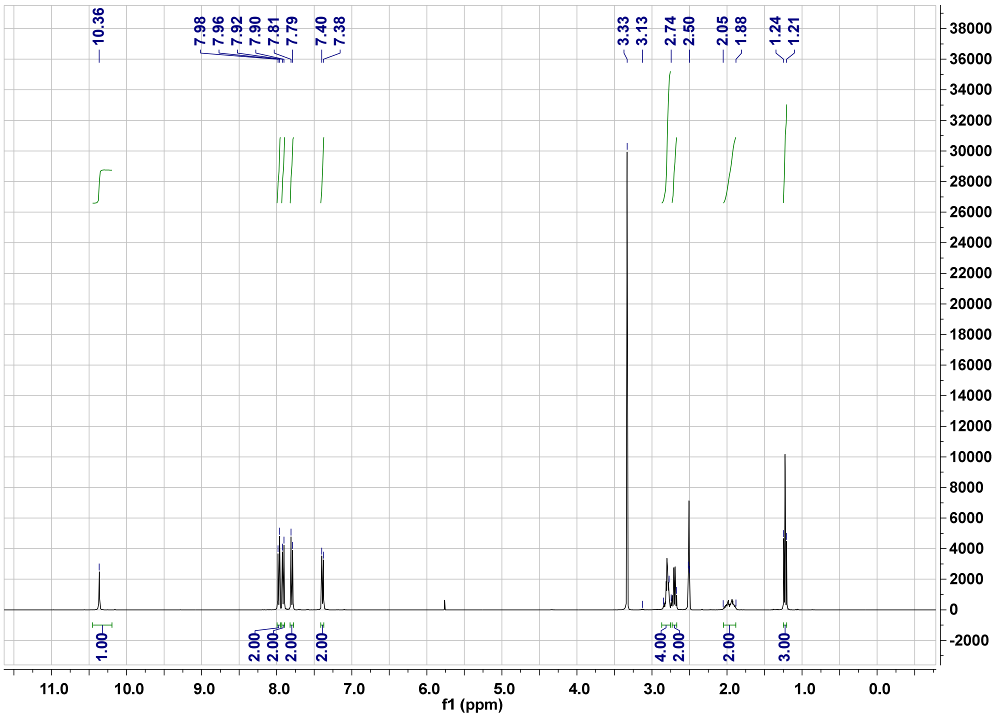
f1 (ppm)

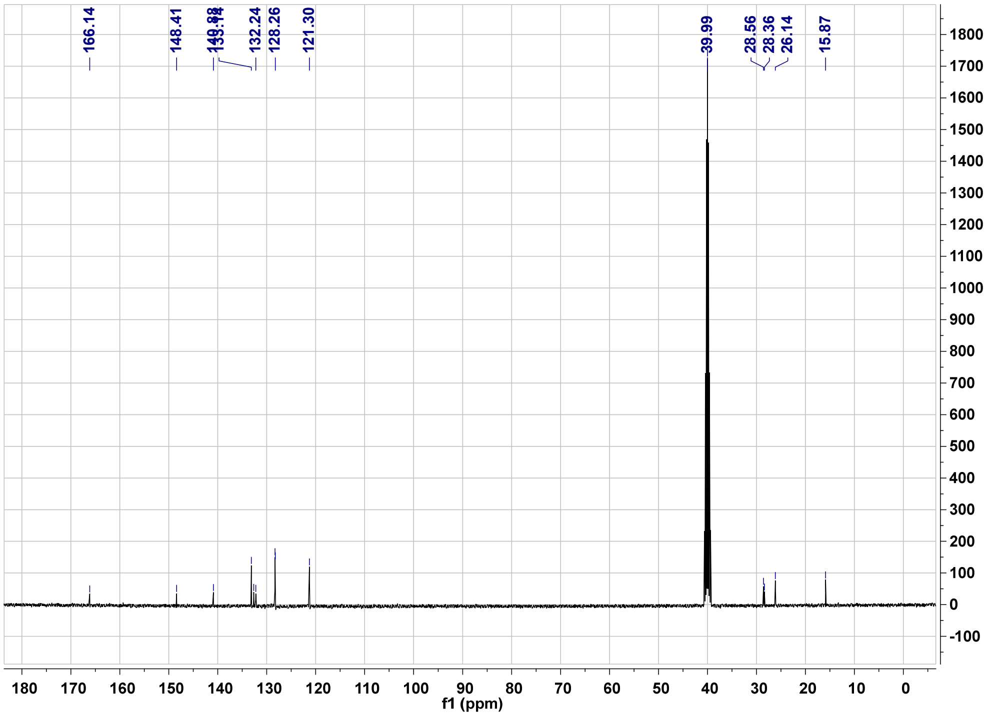

166.14
148.41
132.24
128.26
121.30
39.99
28.56
28.36
26.14
15.87
f1 (ppm)

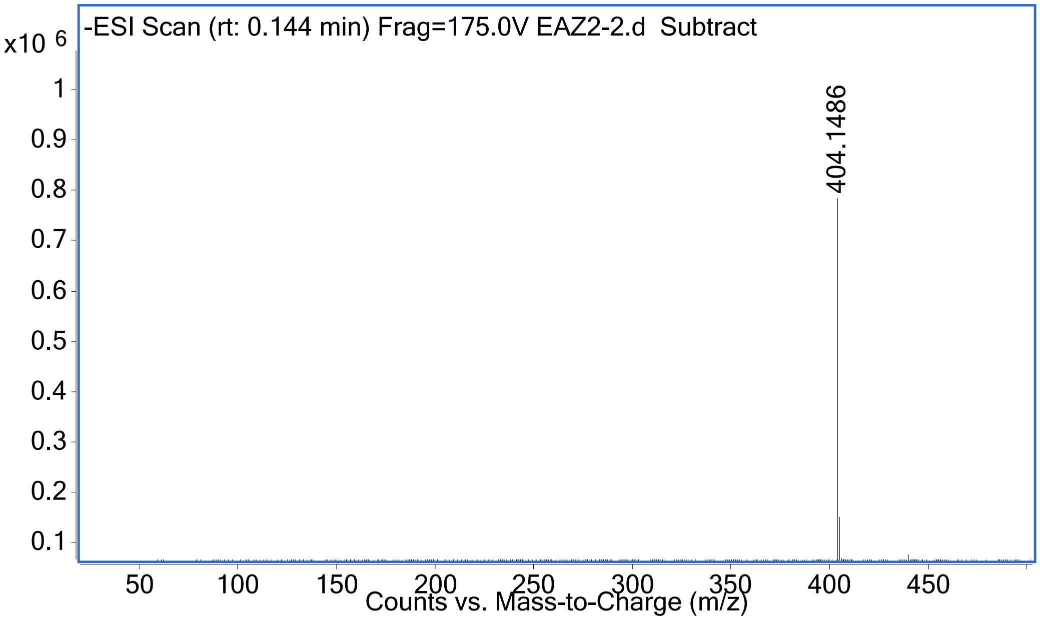

-ESI Scan (rt: 0.144 min) Frag=175.0V EAZ2-2.d Subtract
x10 6
404.1486
Counts vs. Mass-to-Charge (m/z)

AZ2

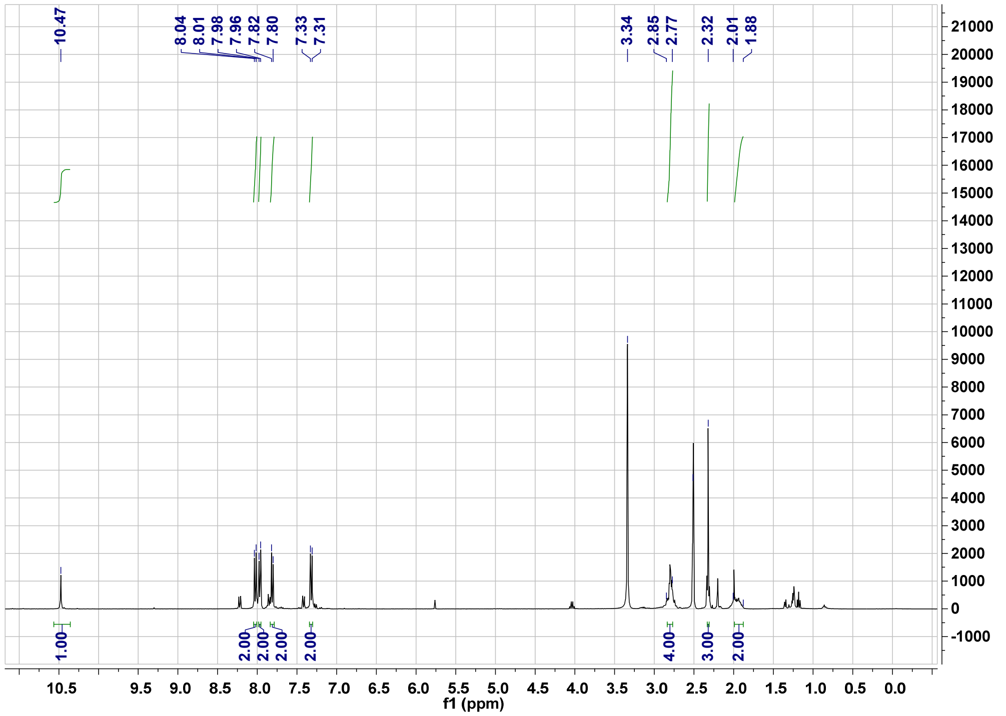
10.47
8.04
8.01
7.98
7.96
7.82
7.80
7.33
7.31
3.34
2.85
2.77
2.32
2.01
1.88
1.00
2.00
2.00
2.00
2.00
4.00
3.00
2.00
f1 (ppm)

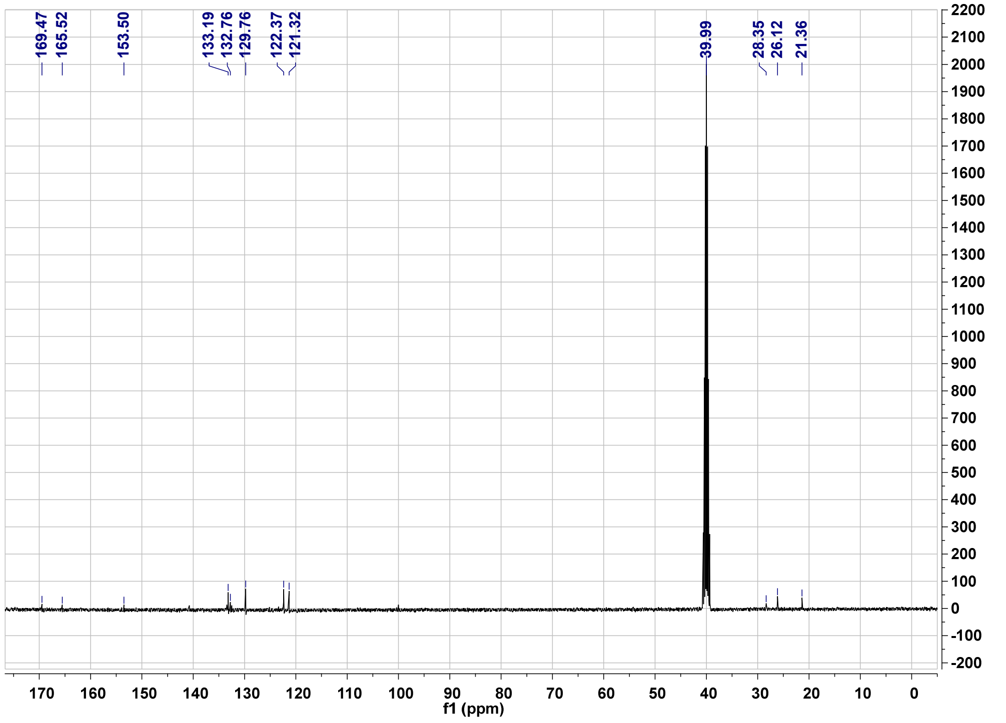
169.47
165.52
153.50
133.19
132.76
129.76
122.37
121.32
39.99
28.35
26.12
21.36
f1 (ppm)

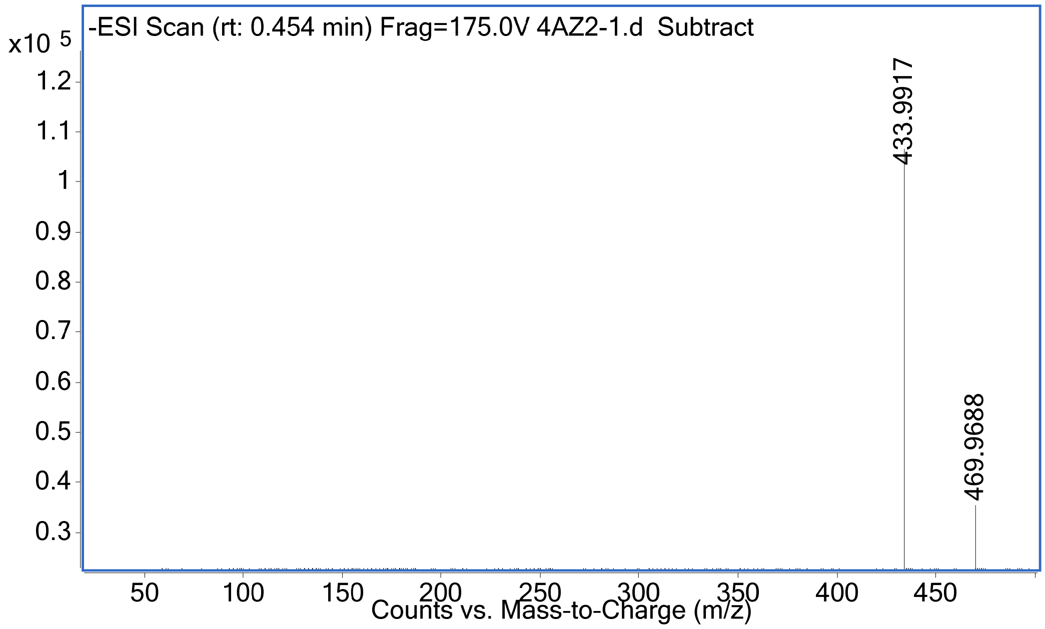

Figure S1. ^{1}H and ^{13}C NMR and Mass spectra of all the synthesized compounds.

MTT assay of arsenicals to 6 cell lines

Table S1. Inhibition effect of organic arsenicals 24 h incubation.

IC_{50}/μM	Molm	NB4	4T1	Hela	COS-7	PBMC
Z2	0.60±0.01	1.50±0.05	1.67±0.14	1.01±0.01	6.1±0.2	4.6±0.3
Z5	0.45±0.01	0.40±0.03	0.87±0.09	0.30±0.13	1.75±0.08	3.5±0.3
PZ2	1.06±0.03	0.85±0.10	2.55±0.22	0.70±0.05	4.1±0.1	6.4±0.2
PZ5	0.27±0.09	0.3±0.03	0.40±0.04	0.26±0.03	1.01±0.02	5.0±0.1
PFZ2	0.80±0.02	0.50±0.02	1.00±0.11	0.50±0.12	6.0±0.1	6.3±0.1
HDZ2	0.60±0.12	0.80±0.11	1.00±0.11	2.0±0.2	9.1±0.2	5.8±0.2
TAZ2	1.60±0.12	1.00±0.11	3.5±0.1	2.0±0.2	9.1±0.15	7.3±0.3
TAZ5	0.71±0.02	0.20±0.03	0.70±0.03	0.80±0.04	2.0±0.1	2.2±0.1
DAZ2	0.90±0.09	1.71±0.12	1.50±0.12	2.3±0.1	7.1±0.1	6.2±0.3
DAZ5	0.45±0.06	0.80±0.01	0.60±0.10	0.81±0.03	1.61±0.09	4.1±0.3
MAZ2	0.20±0.09	0.40±0.12	0.80±0.12	0.40±0.06	5.2±0.1	7.6±0.2
FZ2	1.20±0.11	0.40±0.02	2.3±0.2	1.20±0.10	3.5±0.1	5.5±0.2
FZ5	0.50±0.01	0.15±0.1	0.70±0.02	0.35±0.10	1.10±0.21	2.4±0.2
MZ2	0.20±0.09	0.40±0.12	0.80±0.12	0.40±0.06	5.2±0.1	3.4±0.2
EZ2	1.10±0.10	0.41±0.05	1.40±0.12	1.01±0.12	4.2±0.1	3.7±0.2
AZ2	1.11±0.10	0.60±0.05	2.0±0.1	0.80±0.12	2.2±0.1	5.2±0.3

Table S2. Inhibition effect of organic arsenicals 48 h incubation.

IC_{50}/μM	Molm	NB4	4T1	Hela	COS-7	PBMC
Z2	0.53±0.02	1.20±0.10	1.89±0.22	0.70±0.03	3.2±0.1	4.0±0.2
Z5	0.41±0.02	0.30±0.01	0.84±0.12	0.25±0.20	0.81±0.05	2.8±0.2
PZ2	0.75±0.03	0.70±0.06	3.35±0.42	0.44±0.05	1.75±0.11	5.3±0.3
PZ5	0.24±0.08	0.2±0.02	0.83±0.06	0.14±0.01	0.82±0.04	4.5±0.2
PFZ2	0.70±0.03	0.30±0.01	0.80±0.11	0.15±0.03	3.5±0.1	5.7±0.2
HDZ2	0.60±0.12	0.50±0.05	1.01±0.12	1.01±0.11	3.5±0.1	4.9±0.1
TAZ2	1.50±0.12	0.60±0.05	2.2±0.1	1.04±0.11	5.2±0.11	6.5±0.2
TAZ5	0.70±0.04	0.15±0.01	0.70±0.03	0.50±0.10	1.80±0.12	1.54±0.32
DAZ2	0.75±0.11	1.14±0.09	1.20±0.11	1.06±0.05	3.3±0.2	5.7±0.1
DAZ5	0.32±0.03	0.51±0.08	0.50±0.11	0.55±0.13	1.27±0.15	3.5±0.2
MAZ2	0.10±0.11	0.30±0.09	0.40±0.11	0.30±0.05	3.3±0.2	6.9±0.1
FZ2	1.00±0.11	0.30±0.1	2.2±0.1	0.40±0.05	2.2±0.1	4.7±0.2
FZ5	0.50±0.02	0.15±0.02	0.60±0.01	0.15±0.05	0.80±0.05	1.82±0.12
MZ2	0.10±0.11	0.30±0.09	0.40±0.11	0.30±0.05	3.3±0.2	2.7±0.3
EZ2	1.02±0.02	0.30±0.03	1.40±0.12	0.81±0.11	2.1±0.1	2.9±0.3
AZ2	1.01±0.02	0.60±0.03	1.00±0.12	0.50±0.11	2.1±0.1	4.9±0.3

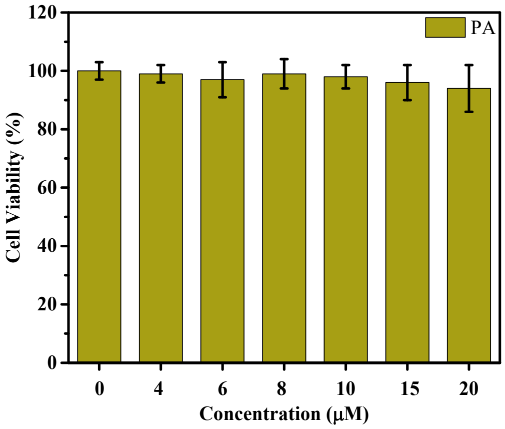

Figure S2. MTT assay of piperonylic acid (PA), precursor of PZ2.

MAZ2 inhibited TrxR activity by binding to the C-terminal Sec/Cys pair

Table S3. The distance of arsenic and active sites of TrxR or Trx, and the binding energy.

			Z2	MAZ2
distance (Å)	TrxR	Cys497	10.6	7.4
		Sec498	6.0	4.7
	Trx	Cys32	9.3	10.1
		Cys35	7.6	8.6
binding energy (kcal/mol)	TrxR		-3.90	-5.20
	Trx		-3.83	-4.12

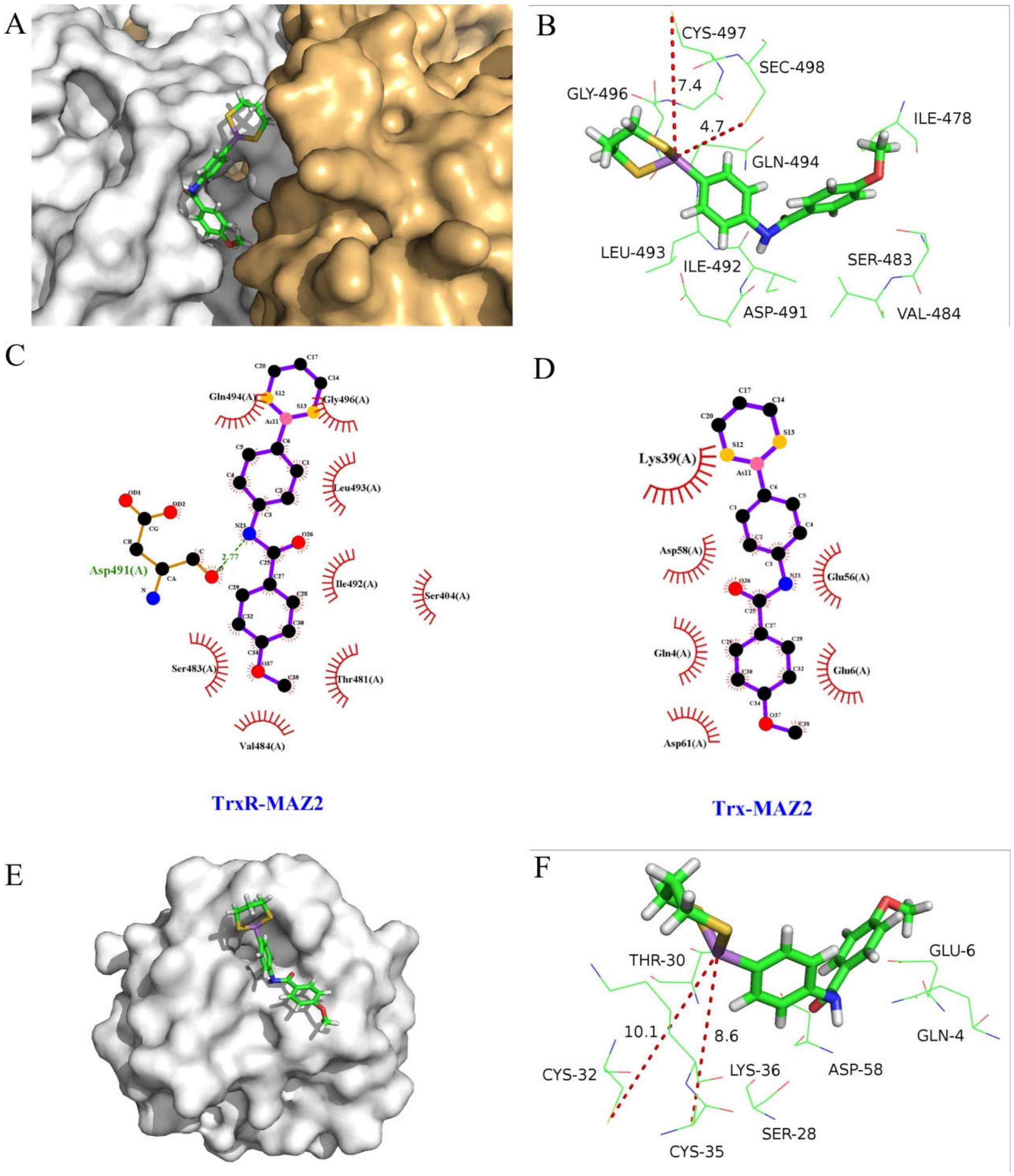

Figure S3. Binding site of MAZ2 in TrxR1 (PDB: 3EAN) and Trx1(PDB: 1ERT).

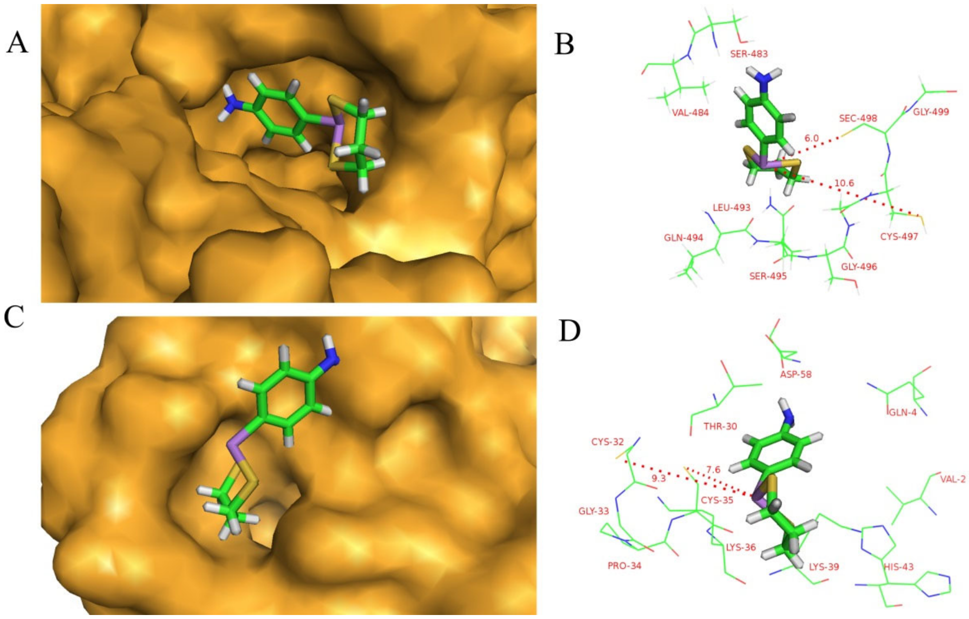

Figure S4. Binding site of Z2 in TrxR1 (PDB: 3EAN) and Trx1(PDB: 1ERT).

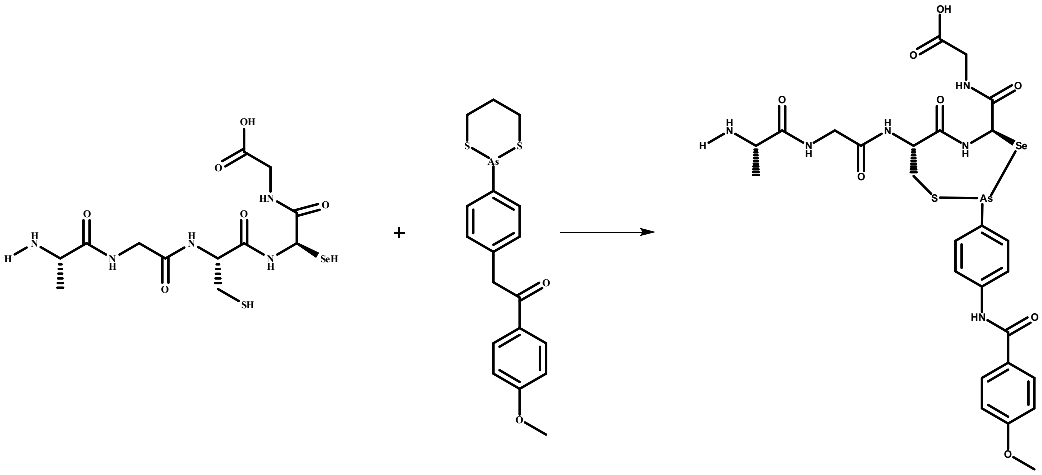

Figure S5. Reaction of AGCUG and MAZ2.

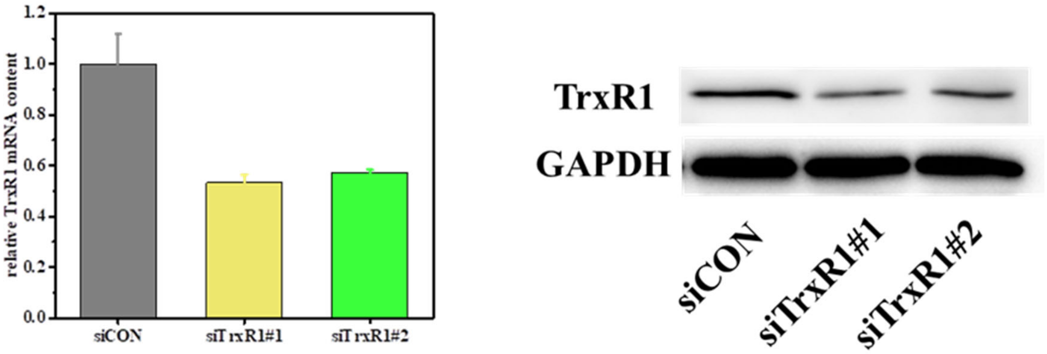

Figure S6. TrxR was knocked down by two siRNA, proved by PCR and WB.

MAZ2 administration had no obvious side effects

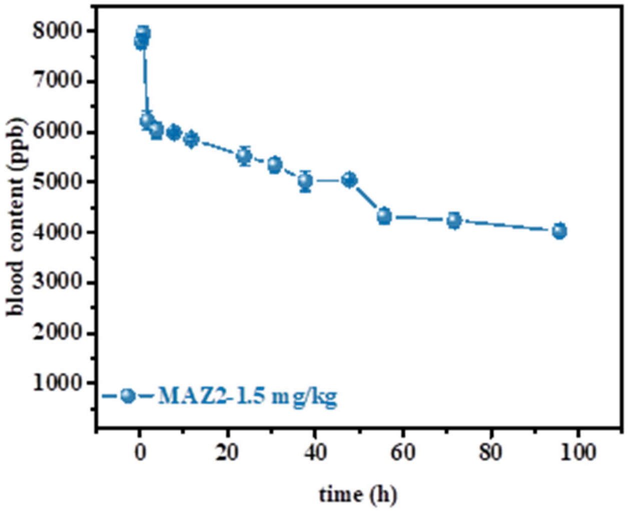

Figure S7. Arsenic content of rat blood administrated with 1.5 mg/kg MAZ2.

siRNA

Table S4. sequence of siRNA.

	sense (5′-3′)	antisense (5′-3′)
TrxR1-#1	CAACCUUAAAGACGAUGAATT	UUCAUCGUCUUUAAGGUUGTT
TrxR1-#2	GGAAGAGAUUCUUGUACGATT	UCGUACAAGAAUCUCUUCCTT

PCR

Table S5. sequence of primer.

Gene	sequences
TrxR1	Forward 5′-TCCACAAACAGCGAGGAGAC -3′<br>Reverse 5′-TGTCACCGATGGCGTAGATG -3′
TrxR2	Forward 5′-AATCTGGAGAAGGCTGGCATC-3′<br>Reverse 5′-TTCCTGCCTTGATAGCTGTGG -3′
Trx1	Forward 5′-ACTTCTCTGCTACGTGGTGTG -3′<br>Reverse 5′-CGGCATGCATTTGACTTCACA -3′
Trx2	Forward 5′-TAACAGTAATGCCCAGCCCAG -3′<br>Reverse 5′-ACCACTGTGCATGAAAGTCCA -3′
β-actin	Forward 5′-GGACTCCTATGTGGGTGACG -3′<br>Reverse 5′-CTTCTCCATGTCGTCCCAGT -3
